# Mechanics-driven nuclear localization of YAP can be reversed by N-cadherin ligation in mesenchymal stem cells

**DOI:** 10.1038/s41467-021-26454-x

**Published:** 2021-10-28

**Authors:** Cheng Zhang, Hongyuan Zhu, Xinru Ren, Bin Gao, Bo Cheng, Shaobao Liu, Baoyong Sha, Zhaoqing Li, Zheng Zhang, Yi Lv, Haohua Wang, Hui Guo, Tian Jian Lu, Feng Xu, Guy M. Genin, Min Lin

**Affiliations:** 1 The Key Laboratory of Biomedical Information Engineering of Ministry of Education, School of Life Science and Technology, Xi’an Jiaotong University, Xi’an, 710049 People’s Republic of China; 2 Bioinspired Engineering and Biomechanics Center (BEBC), Xi’an Jiaotong University, Xi’an, 710049 People’s Republic of China; 3 Department of Endocrinology, Second Affiliated Hospital of Air Force Military Medical University, Xi’an, 710038 People’s Republic of China; 4 State Key Laboratory of Mechanics and Control of Mechanical Structures, Nanjing University of Aeronautics and Astronautics, Nanjing, 210016 People’s Republic of China; 5 School of Basic Medical Science, Xi’an Medical University, Xi’an, 710021 People’s Republic of China; 6 National Local Joint Engineering Research Center for Precision Surgery & Regenerative Medicine, Shaanxi Provincial Center for Regenerative Medicine and Surgical Engineering, The First Affiliated Hospital of Xi’an Jiaotong University, Xian, People’s Republic of China; 7 Department of Medical Oncology, The First Affiliated Hospital of Xi’an Jiaotong University, Xi’an, 710061 Shaanxi People’s Republic of China; 8 MOE Key Laboratory of Multifunctional Materials and Structures, Xi’an Jiaotong University, Xi’an, 710049 People’s Republic of China; 9 Department of Mechanical Engineering & Materials Science, Washington University in St. Louis, St. Louis, 63130 MO USA; 10 NSF Science and Technology Center for Engineering Mechanobiology, Washington University in St. Louis, St. Louis, 63130 MO USA

**Keywords:** Computational biophysics, Biomaterials - cells, Mechanotransduction, Mesenchymal stem cells, Biomaterials - cells

## Abstract

Mesenchymal stem cells adopt differentiation pathways based upon cumulative effects of mechanosensing. A cell’s mechanical microenvironment changes substantially over the course of development, beginning from the early stages in which cells are typically surrounded by other cells and continuing through later stages in which cells are typically surrounded by extracellular matrix. How cells erase the memory of some of these mechanical microenvironments while locking in memory of others is unknown. Here, we develop a material and culture system for modifying and measuring the degree to which cells retain cumulative effects of mechanosensing. Using this system, we discover that effects of the RGD adhesive motif of fibronectin (representative of extracellular matrix), known to impart what is often termed “mechanical memory” in mesenchymal stem cells via nuclear YAP localization, are erased by the HAVDI adhesive motif of the N-cadherin (representative of cell-cell contacts). These effects can be explained by a motor clutch model that relates cellular traction force, nuclear deformation, and resulting nuclear YAP re-localization. Results demonstrate that controlled storage and removal of proteins associated with mechanical memory in mesenchymal stem cells is possible through defined and programmable material systems.

## Abstract (web summary)

Mesenchymal stem cells adopt differentiation pathways based upon mechanical cues in their environment which change throughout development. Here the authors develop a material and culture system to modify and measure the degree to which cells retain cumulative effects of mechanosensing to explore how cells erase the memory of some cues while locking in memory of others.

## Introduction

Human mesenchymal stem cells (hMSCs) differentiate into a range of cell types depending in part upon the mechanical cues they received^1–4^. These cues can vary dramatically during development^5^, healing^6^, cancer^7^ and regenerative therapies^8^, due to stochastic variations, stress concentrations, and cell culture protocols. For example, in regenerative applications^9,10^, MSC populations are typically expanded on stiff (~3 GPa^11^) plastic substrates for relatively long intervals and must forget these mechanical signals prior to transplantation into compliant (~kPa) in vivo microenvironments. Understanding how MSCs learn to forget their previous mechanical microenvironment variations holds potential for improving MSC therapies.

The opposite problem – the ways that MSCs learn and remember their mechanical environments – has been long studied in isolated MSCs. A key indicator of the degree to which MSCs perceive and transduce mechanobiological signals and commit to a lineage-based upon these is the action of the YAP/TAZ transcriptional co-regulators^12–14^. Nuclear compartmentalization of YAP/TAZ in MSCs cultured on relatively stiff substrates activates signaling pathways that promote osteogenesis^15–17^, while cytoplasmic retention of YAP/TAZ in MSCs cultured on relatively compliant substrates promotes adipogenesis^18–20^. Sufficiently long (~10 d) exposure of MSCs to stiff substrates causes nuclear accumulation of YAP/TAZ that is retained to influence long-term cell fate, a phenomenon that is widely termed “mechanical memory”^11,21^. For example, MSCs retain their osteogenic differentiation course when re-exposed to compliant substrates, with the nuclear accumulation of YAP/TAZ becoming apparently irreversible^11^. Several pathways have been identified for this persistence, in addition to the cytoplasm-nucleus shuttling of YAP, including a role for cytoplasmic miR-21 whose presence extends the persistence of the effects of stiff (100 kPa) substrates in MSCs subsequently cultured on soft (15 kPa) hydrogels, and whose knockdown effectively eliminates this persistence^22^.

However, cells, including differentiating MSCs, rarely exist in isolation in vivo^14,23^. Two questions thus arise: how do MSCs know to take cues from their extracellular matrix (ECM) rather than from their compliant neighbors, and can MSCs use information from their neighbors to learn when to forget previous mechanical information? Cell-cell contact affects mechanosensation, with confluent cells that have reached a stable density showing cytoplasmic re-localization of YAP/TAZ^24–26^. Cell-ECM connections (focal adhesions) and cell-cell connections (cadherin junctions) share the same cytoskeletal force network and have many other similarities^27–29^. Sufficiently high adhesion or contractile force in this cytoskeletal network causes YAP to translocate to the nucleus^30,31^. However, there is a trade-off: cell-ECM adhesion weakens cell-cell adhesion, and vice versa^32^, with FAK activation^33^ from strong integrin-ECM connections^34,35^ downregulating VE-cadherin-^36,37^ and N-cadherin-^28,38^ mediated intercellular connections. Adhesion ligation experiments reveal that N-cadherin connections compete with integrin connections to mediate force and determine YAP localization, MSC mechanosensing, and MSC fate^39,40^. N-cadherin clearly affects YAP localization and MSC behavior, but has not been studied in the context of “mechanical memory”.

In this work, we hypothesize that N-cadherin in cell-cell interactions could reverse these effects (“erase” mechanical memory) in MSCs by restoring YAP to the cytoplasm. We thus develop a material system with independently or jointly presented RGD peptides for cell-ECM adhesion and HAVDI peptides for cell-cell adhesion. We harness this to query how these adhesive interactions interact to affect persistent cellular changes in hMSCs in response to mechanosensing, and apply this to control the differentiation of hMSCs.

## Results and discussion

### Physiological responses to cell-cell interactions can be mimicked by HAVDI immobilized on a hydrogel

**Fig. 1:**
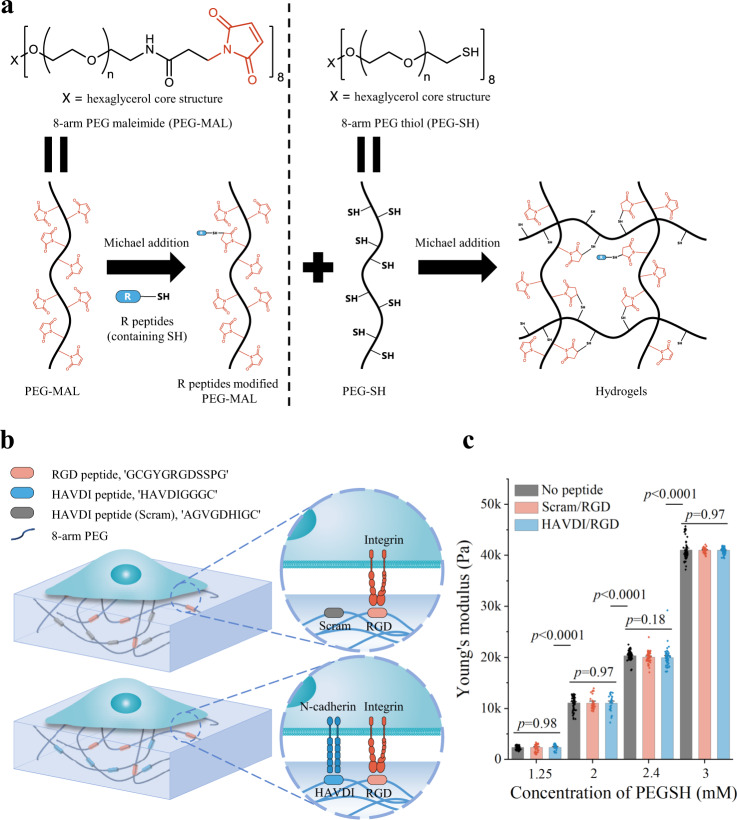
Peptide-functionalized PEG gels that mimic integrin and cadherin adhesion. **a** Mono-thiolated peptides functionalized PEG backbone (8-arm PEG maleimide, PEG-MAL) and subsequent crosslinking with 8-arm PEG thiol (PEG-SH) via Michael addition reaction. **b** Schematic of PEG-MAL hydrogels modified with HAVDI and RGD peptides acting as N-cadherin and fibronectin adhesive domains, respectively. Scram/RGD hydrogel substrate allows only integrin/RGD interactions, while HAVDI/RGD substrate allows both integrin/RGD and N-cadherin/HAVDI interactions. **c** Young’s moduli of hydrogels synthesized at different PEGSH concentrations without peptide (No peptide) or with peptide (Scram/RGD or HAVDI/RGD). Results indicated that modification of peptides did not alter the stiffness of PEG hydrogels in this system. The data represented the mean ± s.e.m., from left to right, *n* = 60, 66, 47, 31, 60, 30, 30, 89, 50, 50, 60, 46, 65 points respectively, adopted from 3 independent samples for each group (*p* values were obtained using one-way ANOVA followed by Tukey’s post hoc test, mean ± s.e.m.). Source data are provided as a Source Data file.

To elucidate how N-cadherin affects YAP localization, we developed a dual-peptide functionalized PEG hydrogel analogous to the hyaluronic acid (HA) based hydrogel of Cosgrove et al.^39^. The PEG hydrogel system presented either HAVDI/RGD (from N-cadherin, representing neighboring cells), or non-functional scrambled HAVDI sequence (Scram) combined with RGD (Scram/RGD) (Fig. 1). 8-arm PEG maleimide (PEG-MAL) modified with mono-thiolated peptides was subsequently crosslinked by 8-arm PEG thiol (PEG-SH) via Michael addition) (Fig. 1a)^41^. As with analogous HA hydrogels, PEG hydrogels modified with 1 mM RGD and 1 mM non-functional scrambled HAVDI sequence (Scram/RGD) established only integrin adhesions, while PEG hydrogels modified with 1 mM RGD and 1 mM HAVDI enabled both integrin and N-cadherin mediated interactions (HAVDI/RGD) (Fig. 1b and Supplementary Fig. 1). Concentrations of HAVDI peptides followed Cosgrove et al.^39^, and were within the range of E-cadherin density at adherens junctions in monolayer epithelial cells of developing Drosophila embryos^42^. The grafted peptide was observed to distribute homogeneously on the surface of hydrogels (Supplementary Fig. 1). The binding efficiency of peptide to the PEG backbone was measured at over 86% (Supplementary Fig. 2). Through the binding efficiency, we calculated the surface concentrations of peptides, which fell into a reasonable range (Supplementary Text).

Hydrogel stiffness could be tuned independently from 2-41 kPa by varying relative concentrations of PEG-SH and PEG-MAL (Fig. 1c). Modification with HAVDI/RGD or Scram/RGD did not affect Young’s moduli of the hydrogel over the range of concentrations of PEG-SH and PEG-MAL used in this study (Fig. 1c). Young’s moduli were obtained from load-indentation curves (Supplementary Fig. 3). When we seeded cells on the hydrogels with different stiffness and peptide, cell area increased with increasing substrate stiffness, in accordance with previous studies^43,44^; no significant difference was observed between the Scram/RGD and HAVDI/RGD groups (Supplementary Fig. 4). Results indicated that the HAVDI peptide did not affect cell spreading, and differences in YAP n/c ratio observed between Scram/RGD and HAVDI/RGD hydrogels did not arise from difference in cell area.

**Fig. 2:**
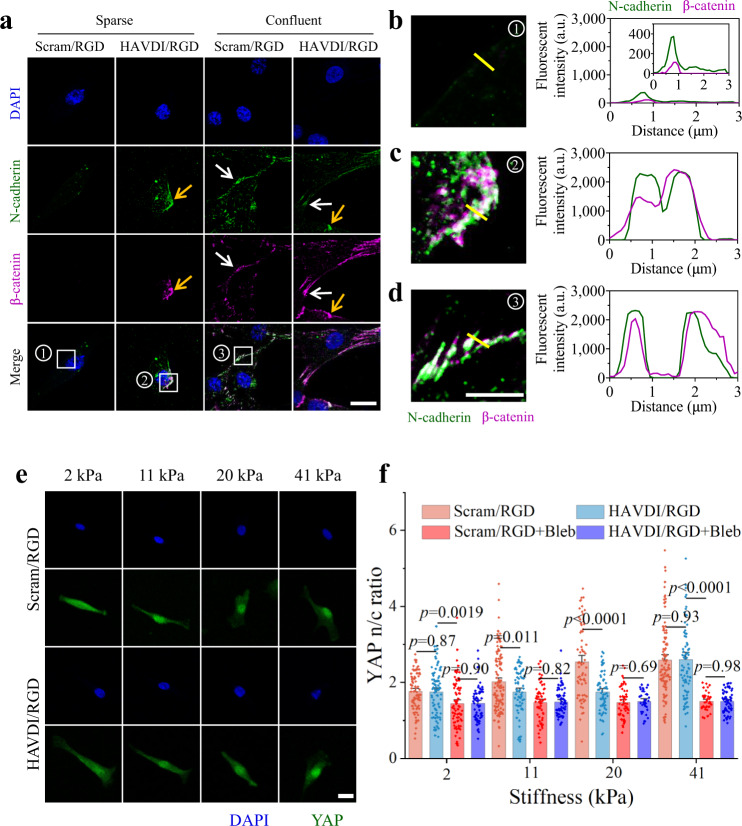
N-cadherin clustering and β-catenin recruitment in mimetic PEG hydrogels and stiffness dependent YAP nuclear localization via N-cadherin signaling. **a** HAVDI ligation caused sparse cells to adopt the N-cadherin and β-catenin patterning of confluent cells, as evident from confocal images of DAPI (blue), N-cadherin (green) and β-catenin (purple) staining in hMSCs cultured on either sparse (non-confluent) or confluent conditions on Scram/RGD or HAVDI/RGD hydrogels of Young’s modulus 20 kPa. The white arrow highlights real cell-cell adhesions between neighboring hMSCs; the yellow arrow highlights N-cadherin/HAVDI binding for an isolated hMSC on a HAVDI/RGD hydrogel. Scale bar: 20 μm. **b**–**d** Representative subcellular distributions of N-cadherin and β-catenin in three different cases corresponding to the numbered areas shown in **a**, showing that HAVDI ligation caused sparse cells to adopt the N-cadherin and β-catenin patterning of confluent cells. Scale bars: 5 μm. **b** Isolated cells on Scram/RGD hydrogels, which do not mimic cell-cell adhesions, did not show substantial N-cadherin clustering or β-catenin recruitment. **c** Isolated cells cultured on HAVDI/RGD hydrogel, which mimicked cell-cell adhesions, showed N-cadherin clustering or β-catenin recruitment that was indistinguishable from that in **d** confluent cells on Scram/RGD hydrogels. Yellow lines in **b**–**d** indicate the pixel regions of interest used to generate intensity profiles of N-cadherin (green) and β-catenin (purple). **e** Confocal images of YAP immunostaining on hMSCs cultured on 2, 11, 20 and 41 kPa Scram/RGD or HAVDI/RGD hydrogels for 3 d. Scale bars, 20 μm. **f** Quantification of YAP n/c ratios on Scram/RGD or HAVDI/RGD hydrogels of increasing stiffness with or without blebbistatin (myosin inhibitor) as shown in **e** (from left to right *n=* 82, 87, 79, 74, 126, 71, 60, 65, 71, 68, 53, 35, 105, 82, 39, 63 cells examined over 14, 13, 16, 18, 17, 16, 27, 19, 15, 13, 19, 24, 9, 9, 23, 27 images respectively, *p* values were obtained using one-way ANOVA followed by Tukey’s post hoc test, mean ± s.e.m.). Source data are provided as a Source Data file.

We began by using this system to establish whether HAVDI presentation faithfully replicates the N-cadherin clustering and β-catenin recruitment associated with N-cadherin-mediated cell-cell interactions. These two mechanosensing proteins accumulate as force transmission between cells matures adherens junctions via an α-catenin/vinculin dependent pathway^45^. To study this, we cultured hMSCs at prescribed cell densities and levels of confluence on Scram/RGD or HAVDI/RGD hydrogels with a stiffness of 20 kPa for 3 d, then fixed and stained the hMSCs for N-cadherin and β-catenin on their basal planes (Fig. 2a).

Sparse cells that had no contact with their neighbors showed neither N-cadherin clustering nor β-catenin recruitment when cultured on Scram/RGD hydrogels (Fig. 2b). However, N-cadherin clustering and β-catenin co-localization were rescued in sparse cells cultured upon HAVDI/RGD hydrogels (Fig. 2c). Likewise, confluent cells, which formed adherens junctions with their neighbors, showed clustering of N-cadherin as well as recruitment and co-localization of β-catenin on both the HAVDI/RGD and Scram/RGD hydrogels (Fig. 2d). To check whether N-cadherin co-localized with β-catenin, the percentage of N-cadherin area that was also positive for β-catenin was quantified from fluorescence images using a custom MATLAB (R2014a) script that implemented standard cross-correlation techniques from the literature^39,46^ (Supplementary Fig. 5). For both sparse and confluent cells, significantly more co-localization (*i.e*., N-cadherin area positive for β-catenin) was observed for cells cultured on HAVDI/RGD hydrogels than for cells cultured on Scram/RGD hydrogels, indicating that substrates with immobilized HAVDI induced both N-cadherin clustering and β-catenin recruitment.

Treatment with 1 mM soluble HAVDI(-C) peptide, designed to bind with membrane N-cadherin receptors while avoiding conjugation to the PEG-Mal backbone, blocked recruitment and co-localization of N-cadherin and β-catenin in cells cultured on both Scram/RGD and HAVDI/RGD hydrogels (Supplementary Fig. 6). These results suggested that HAVDI/N-cadherin ligation arising in cells cultured on HAVDI/RGD hydrogels served to generate force from substrates triggering recruitment and co-localization β-catenin forming force bearing proteins chain.

### HAVDI ligation reverses YAP nuclear localization in hMSCs

HAVDI presentation is known to affect nuclear localization of YAP^39^. Immunostaining that replicated this result in our PEG system showed nucleus-to-cytoplasm (n/c) ratios of YAP significantly attenuated by HAVDI presentation over an intermediate range of substrate moduli (10~20 kPa), but unaltered for compliant (~1 kPa) or stiff (~40 kPa) substrates (Fig. 2e–f). Consistent with the observations of others^15,39,47–50^, our myosin inactivation experiments showed YAP signaling to be highly dependent on the contractile state of the cell (Fig. 2f). After myosin inactivation using blebbistatin, we found that all YAP n/c ratios dropped to the same level for both the Scram/RGD and HAVDI/RGD hydrogels, independent of substrate moduli (2-41 kPa). These results suggested that actomyosin inhibition shielded the mechanosensing of substrates stiffness by cells^48–50^. In addition, differences of YAP n/c ratio on substrates with different peptides could be attributed to different actomyosin contractility, because myosin inactivation could eliminate the observed HAVDI effect.

To quantify how peptide concentrations affect nuclear localization of YAP, we varied the HAVDI/Scram/RGD peptide concentration ratios on hydrogels while keeping the total effective ligand density constant. Increasing RGD presentation resulted in un-changed YAP nuclear-to-cytoplasmic (n/c) ratios on Scram/RGD hydrogels, indicating that 0.25 mM RGD was sufficient to induce a maximal YAP n/c ratio (Supplementary Fig. 7). Cells on HAVDI/RGD hydrogels showed lower YAP n/c ratios, with the differential becoming more pronounced with increasing HAVDI concentration. These results suggested that the increasing RGD concentration (≥0.25 mM) did not affect YAP nuclear translocation measurably, while increasing HAVDI concentration inhibited it.

We therefore chose two hydrogels with a fixed, intermediate stiffness (Young’s modulus = 20 kPa) and two different peptide combinations (Scram/RGD for 1 mM Scram and 1 mM RGD peptides, HAVDI/RGD for 1 mM HAVDI and 1 mM RGD peptides) to test the hypothesis that N-cadherin ligation can reverse nucleus-to-cytoplasm shuttling of YAP.

Before studying this reversal of nucleus-to-cytoplasm shuttling of YAP, we repeated earlier results showing that sparse hMSCs developed significantly higher YAP nuclear localization when cultured on Scram/RGD hydrogels than when cultured on HAVDI/RGD hydrogels (Supplementary Fig. 8a, upper-left; Supplementary Fig. 8b, left); blocking N-cadherin with soluble HAVDI(-C) peptide (1 mM) restored the YAP n/c ratio in sparse hMSCs cultured on HAVDI/RGD substrates to the level seen in hMSCs cultured on Scram/RGD substrates (Supplementary Fig. 8a, lower-left; Supplementary Fig. 8b, left), consistent with the observations of Cosgrove et al.^39^. YAP was excluded from the nucleus in hMSCs in confluent cells cultured on either Scram/RGD or HAVDI/RGD substrates (Supplementary Fig. 8a, upper-right; Supplementary Fig. 8b, right), consistent with previous studies^15,18,25^ and suggesting that cell-cell interactions inhibited nuclear localization of YAP. Blocking N-cadherin with soluble HAVDI(-C) peptide abrogated this inhibition in confluent cells as well (Supplementary Fig. 8a, lower-right; Supplementary Fig. 8b, right), indicating that N-cadherin mechanoresponsiveness controlled YAP exclusion associated with both real and mimicked cell-cell interactions.

**Fig. 3:**
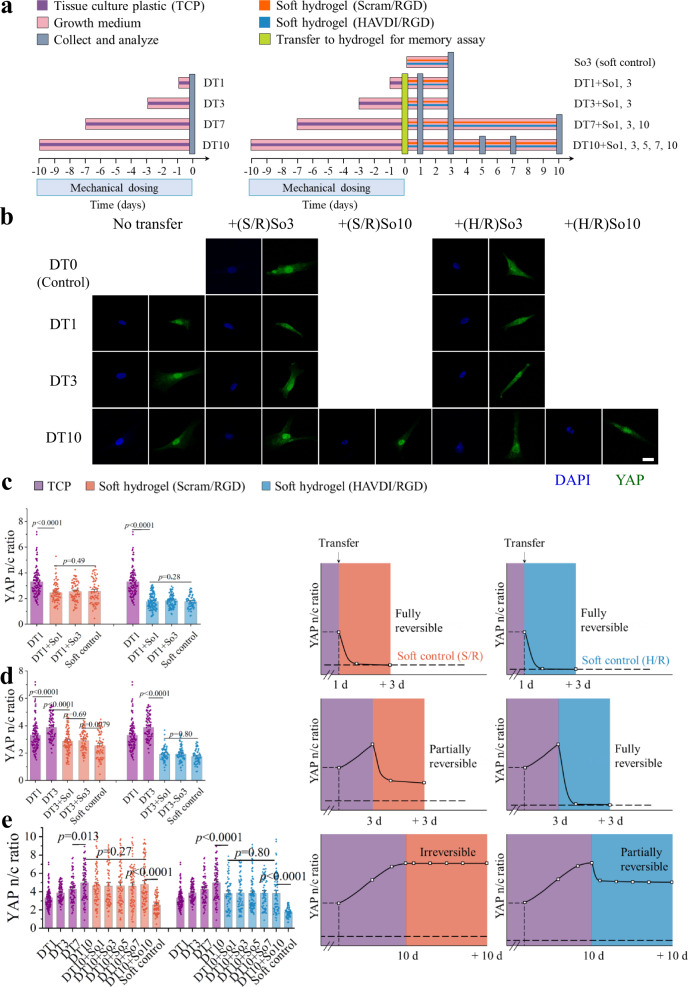
HAVDI ligation reversed YAP nuclear localization in hMSCs. **a** Schematic of the experimental protocol for assessing the role of HAVDI on reversing YAP nuclear localization in hMSCs. YAP accumulation in the nucleus was measured in hMSCs cultured on TCP (dark purple) in growth medium (pink) for 1, 3, 7, or 10 d, denoted DT1, DT3, DT7, and DT10, respectively. Reversal of YAP nuclear accumulation was assessed in hMSCs that were cultured on TCP in growth medium for these same time intervals, but then transferred (light yellow, day 0) to either soft (20 kPa) Scram/RGD (orange) or soft (20 kPa) HAVDI/RGD (blue) hydrogels for 1-10 d in growth medium before collection and analysis (gray, day 1–10). These conditions were denoted (DT1 + So1, 3), (DT3 + So1, 3), (DT7 + So1, 3, 10), or (DT10 + So1, 3, 5, 7, 10). Control cases included 3 d of cell culture on soft Scram/RGD (orange) or HAVDI/RGD (blue) hydrogels (So3), denoted “soft control (S/R)” and “soft control (H/R)”, respectively. **b** Representative immunostaining images of hMSC nuclei (blue) and YAP (green) after specified treatments on stiff and soft substrates as shown in **a**. Scale bars, 20 µm. S/R is Scram/RGD, H/R is HAVDI/RGD. **c**–**e** Quantification of YAP nuclear/cytoplasmic (n/c) ratios in hMSCs after specified treatments on stiff and soft substrates as shown in **a**. S/R (orange) and H/R (blue) represent all conditions related to soft Scram/RGD and HAVDI/RGD hydrogels, respectively. Soft control (S/R) and soft control (H/R) are baselines of YAP n/c ratios after 3 d culture for the S/R and H/R groups, respectively. YAP n/c ratio increased with culture time on TCP. **c** After 1 d of culture on TCP (purple, DT1), YAP localized to the nucleus (from left to right *n =* 108, 79, 70, 71, 108, 120, 100, 68 cells examined over 5, 11, 23, 15, 5, 26, 13, 13 images respectively, *p* values were obtained using one-way ANOVA followed by Tukey’s post hoc test, mean ± s.e.m.). Following transfer of hMSCs to either soft Scram/RGD (orange) or soft HAVDI/RGD (blue) substrates and culture for 1 d or 3 days (DT1 + So1, 3), the YAP n/c ratios decreased separately to the baseline of soft control S/R or H/R levels, showing a fully reversible YAP nuclear accumulation. **d** Following 3 d of culture on TCP (DT3), the YAP n/c ratio remained above the baselines of soft control in hMSCs transferred to Scram/RGD substrates for 1 d or 3 d (S/R: DT3 + So1, 3), but returned to the baselines in hMSCs transferred to HAVDI/RGD substrates for 1 d or 3 d (H/R: DT3 + So1, 3) (from left to right *n =* 108, 85, 93, 77, 71, 108, 85, 68, 72, 68 cells examined over 5, 6, 17, 15, 15, 5, 6, 19, 6, 13 images respectively, *p* values were obtained using one-way ANOVA followed by Tukey’s post hoc test, mean ± s.e.m.). This showed that, following 3 d of culture on TCP, YAP nuclear localization was partially reversible response without HAVDI (S/R), but fully reversible with HAVDI (H/R). **e** Following 10 d of culture on TCP (DT10), the YAP n/c ratio remained elevated and unchanged in hMSCs transferred to Scram/RGD substrates for up to 10 d (S/R: DT10 + So1, 3, 5, 7, 10), but decreased towards baseline levels within 1 d and maintained the levels in hMSCs transferred to HAVDI/RGD substrates for up to 10 d (H/R: DT10 + So1, 3, 5, 7, 10) (from left to right *n =* 108, 85, 66, 76, 74, 66, 78, 84, 84, 71, 108, 85, 66, 76, 70, 75, 102, 70, 78, 68 cells examined over 5, 6, 9, 11, 16, 21, 21, 23, 26, 15, 5, 6, 9, 11, 28, 21, 37, 20, 8, 13 images respectively, *p* values were obtained using one-way ANOVA followed by Tukey’s post hoc test, mean ± s.e.m.). This showed that, following 10 d of culture on TCP, YAP nuclear accumulation was permanent (irreversible) over the experimental timescale without HAVDI (S/R), but partially reversible with HAVDI (H/R). Source data are provided as a Source Data file.

We next tested the hypothesis that YAP nuclear localization, known to arise from priming of cells on stiff tissue culture plastic (TCP, ~3 GPa), could be reversed by N-cadherin signaling from HAVDI/RGD substrates. For this, hMSCs were cultured on TCP for 1-10 d, and then transferred to soft (20 kPa) HAVDI/RGD or Scram/RGD hydrogels for 3 or 10 d (Fig. 3a). As seen by others^11^, the YAP n/c ratio in hMSCs increased with increasing culture time on TCP (Fig. 3b, “no transfer”). When hMSCs were transferred to Scram/RGD or HAVDI/RGD hydrogels after 1 d on TCP substrate (DT1), the YAP n/c ratio returned to the levels seen in cells that had never been exposed to TCP (“soft control” group), which suggested that YAP nuclear localization was fully reversible over 1 d (DT1 + So1) (Fig. 3c). Longer culture time on soft gels (DT1 + So3) did not change the YAP n/c ratio, suggesting that the kinetics of YAP nuclear localization reached a steady state within 1 d.

This observation was in accordance with previous studies that after substrate softening, the YAP n/c ratio reduced to baseline levels within 1 d and maintained these levels for up to 10 d^21^. Cui et al. found that nuclear accumulation of YAP reached a steady state within 6 h^51^, and Elosegui-Artola et al. found that YAP n/c ratio changed within several minutes after nuclear force is applied^31^. To exclude the possible effects of mechanical stimuli generated by cell passaging, we conducted experiments where cells were transferred from TCP to TCP. The YAP n/c ratio showed no difference between groups that had undergone passaging (DT3 + DT0.25 and DT3 + DT3 in Supplementary Fig. 9) and those that had not (DT3.25 and DT6 in Supplementary Fig. 9), indicating that mechanical stimuli associated with transferring substrates had no measurable effect on the MSC mechanosensing or memory.

In hMSCs cultured for 3 d on TCP (DT3) and subsequently transferred to soft HAVDI/RGD hydrogels for 1 d (DT3 + So1), the YAP n/c ratio recovered to the level of “soft control” group. Further increasing the culture time on soft HAVDI/RGD hydrogels to 3 d did not change the YAP n/c ratio. This suggested that YAP nuclear localization was again fully reversed within 1 d (Fig. 3d). In contrast, transferring to Scram/RGD hydrogels led to a higher YAP n/c ratio than that observed in the “soft control” group, suggesting that YAP nuclear localization was only partially reversible under these conditions (Fig. 3d). After 7 d on TCP (DT7), YAP nuclear localization was partially reversible in cells transferred to either Scram/RGD or HAVDI/RGD hydrogels; the YAP n/c ratio of cells transferred to HAVDI/RGD gels dropped more than those of cells transferred to Scram/RGD gels (Supplementary Fig. 10).

In hMSCs cultured for 10 d on TCP (DT10) and then transferred to Scram/RGD hydrogels, the YAP n/c ratio remained the much higher levels in cells in the “soft control” group with 1-10 d culture time. However, transferring to HAVDI/RGD hydrogels led to a drop of the YAP n/c ratio within 1 d to a level that remained constant for up to 10 d culture time. This confirmed that 10 d of culture on TCP made YAP nuclear localization irreversible in cells transferred to soft Scram/RGD hydrogels, as observed in previous studies^11,21^, and revealed that YAP nuclear localization was again partially reversible in hMSCs transferred to HAVDI/RGD hydrogels (Fig. 3e). Increasing HAVDI concentration increased the magnitude of the drop in YAP n/c ratio (Supplementary Fig. 11).

Thus, hMSCs integrated and stored nuclear YAP when exposed to stiff TCP. This was increasingly persistent in response to increasing mechanical dosing on TCP, but could be partially or entirely reversed via HAVDI ligation that mimicked N-cadherin signaling in cell-cell interactions.

### HAVDI ligation can be used to control stem cell fate

**Fig. 4:**
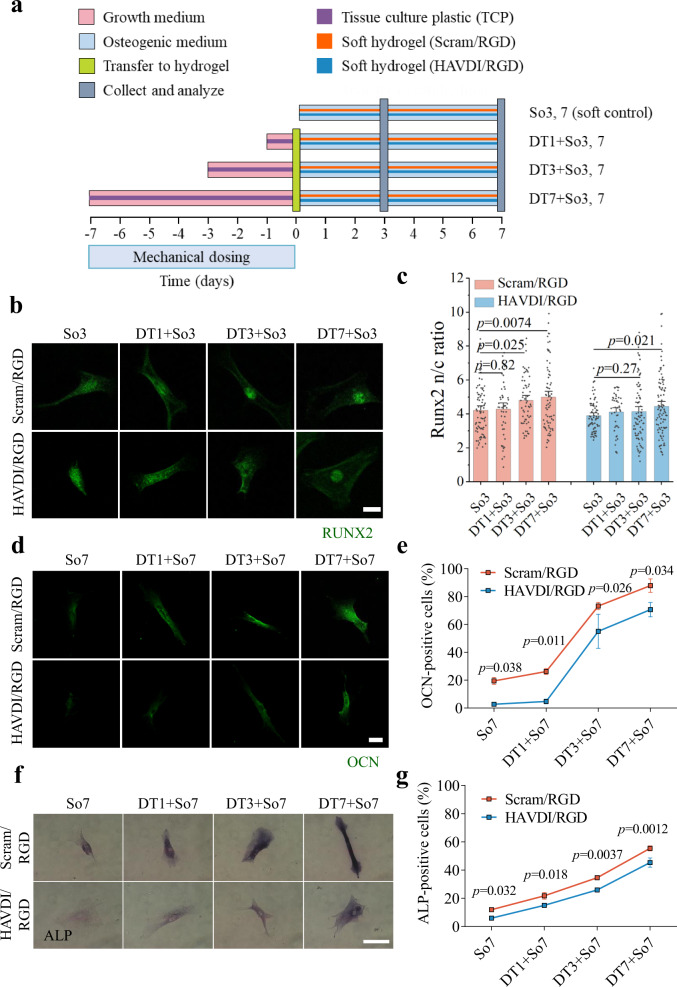
The HAVDI/RGD hydrogel can be used to reduce long-term retention of the effects of mechanotransduction and inhibit osteogenic differentiation of hMSCs. **a** Schematic of the experimental protocol for assessing osteogenic differentiation of hMSCs cultured on TCP for 1-7 d, followed by 3 d (for RUNX2) or 7 d (for OCN and ALP) culture on 20 kPa Scram/RGD or HAVDI/RGD hydrogels. hMSCs were cultured on TCP (dark purple) in growth medium (pink) for 1, 3, or 7 d and then transferred (light yellow, day 0) to soft Scram/RGD (orange) or HAVDI/RGD (blue) hydrogels for additional 3 d or 7 d of culture in osteogenic medium (light blue) before collection and analysis (gray, day 3 or day 7). RUNX2 expression was assessed in the conditions denoted So3 (soft control, without culture on TCP), DT1 + So3, DT3 + So3, or DT7 + So3. OCN and ALP expression were assessed in the conditions denoted So7 (soft control, without culture on TCP), DT1 + So7, DT3 + So7, or DT7 + So7. **b** Representative confocal images of cells stained for RUNX2, a marker of early osteogenic differentiation, in hMSCs following treatment as shown in **a**. Scale bars, 20 µm. **c** Analysis of confocal images showed a significant increase in the RUNX2 n/c ratio with increasing exposure to TCP in hMSCs transferred to Scram/RGD hydrogels (from left to right *n =* 77, 49, 59, 73, 72, 44, 84, 92 cells examined over 7, 15, 19, 20, 12, 24, 23, 26 images respectively, *p*-values were obtained using one-way ANOVA followed by Tukey’s post hoc test, mean ± s.e.m.). However, effects of TCP were evident only after 7 days of exposure to TCP in hMSCs transferred to HAVDI/RGD hydrogels, and was greatly attenuated compared to Scram/RGD for 7 days of mechanical dosing on TCP. **d**–**g** Representative images of staining for late osteogenic differentiation markers OCN (**d**) and ALP (**f**) in hMSCs following the treatment shown in **a**. Scale bars, 20 µm. Quantification of the percentage of OCN-positive cells (**e**) and ALP-positive cells (**g**) affirmed that HAVDI ligation significantly attenuated osteogenic differentiation after mechanical priming on TCPS (*n =* 3 experiments per group, *p* values were obtained using one-way ANOVA followed by Tukey’s post hoc test, mean ± s.e.m.). Source data are provided as a Source Data file.

To demonstrate therapeutically useful reversal of YAP nuclear localization in hMSCs, we explored whether the seemingly irreversible, osteogenic commitment of hMSCs^11^ could be reversed by HAVDI ligation. Yang, et al., demonstrated that priming of hMSCs for 10 d on stiff substrates resulted in nuclear localization of the osteogenic marker RUNX2 even after in-situ softening of the substratum for 10 d^11^. We found similar results for cells cultured in growth medium for 1-7 d on TCP (~3 GPa) then transferred to soft (20 kPa) Scram/RGD hydrogels for 3 d in osteogenic medium (Fig. 4a), with nuclear localization of RUNX2 increasing with increasing culture time on TCP (Fig. 4b, c). However, in cells that were instead transferred from TCP to soft (20 kPa) HAVDI/RGD hydrogels, a negligible increase of RUNX2 nuclear localization was observed following stiff priming of up to 3 d, and elevation of RUNX2 levels was greatly attenuated for stiff priming of 7 d.

To verify that the observed RUNX2 nuclear localization was indicative of osteogenesis, the late osteogenic markers alkaline phosphatase (ALP) and osteocalcin (OCN)^52,53^ were tracked in hMSCs that were exposed to TCP for 1-7 d in growth medium, and that were then transferred onto soft (20 kPa) Scram/RGD or HAVDI/RGD hydrogels for 7 d in osteogenic differentiation medium before being fixed and stained (Fig. 4a). Consistent with the results of Yang, et al.^11^, ALP and OCN expression increased with increasing culture time on TCP for hMSCs transferred to Scram/RGD hydrogels (Fig. 4d–g). However, HAVDI ligation significantly attenuated both ALP and OCN levels, verifying that HAVDI ligation diminished osteogenic commitment. These findings demonstrated HAVDI ligation as a tool for attenuating nuclear localization of YAP and sustaining stem cell plasticity during in vitro expansion.

### HAVDI dependent motor-clutch dynamics affect the mechanical sensation and nuclear translocation of YAP

Growing evidence implicates a motor-clutch machine of myosin, actin filaments, and focal adhesion complexes^54,55^ in sensation of extracellular matrix (ECM) stiffness^56–58^. The forces associated with actomyosin contraction increase with substrate stiffness because of increased resistance to contraction, increased engagement of the motor-clutch mechanism, and increased development of polarized stress fibers. The forces associated with increased traction on the substrate also deform the nucleus and can initiate nuclear-cytoplasmic shuttling of YAP and downstream signaling pathways^31,59^. We hypothesized that N-cadherin affected mechanical sensation and the YAP n/c ratio (${R}_{{{{{{\rm{NC}}}}}}}$) by inhibiting the rate ${k}_{{{{{{\rm{on}}}}}}}$ of integrin binding, and hence reducing integrin clustering, tractions, and nuclear deformation. To test this hypothesis about the effects of HAVDI ligation, we applied a motor-clutch model (model details in Supplementary Methods and Supplementary Table 1)^56–58,60^.

**Fig. 5:**
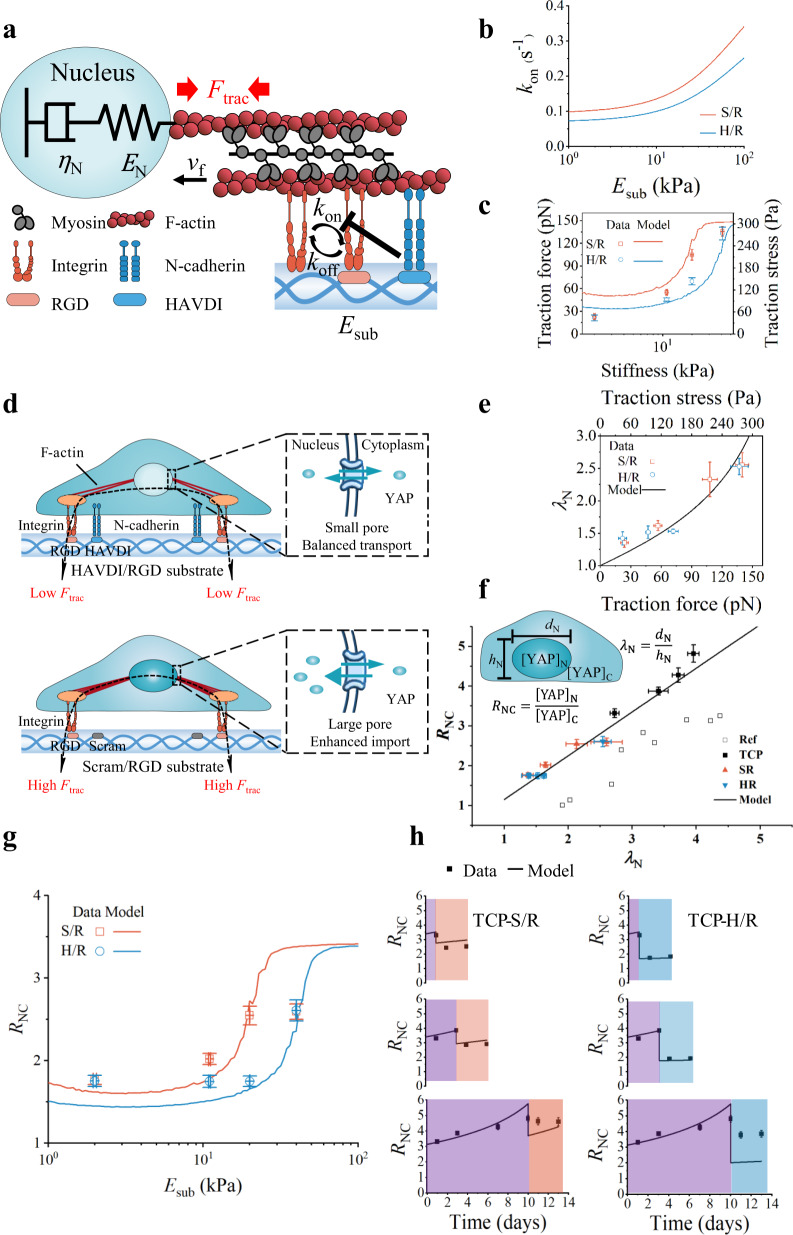
A modified motor-clutch model explains the role of HAVDI ligation in reversing YAP nuclear localization in MSCs. **a** In the motor-clutch model, integrin clutches connected to RGD on ECMs with binding rate (${k}_{{{{{{\rm{on}}}}}}}$), and broke with ECM at force dependent unbinding rate (${k}_{{{{{{\rm{off}}}}}}}$). Myosin motors pulled actin filaments with traction force (${F}_{{{{{{\rm{trac}}}}}}}$) at velocity (${v}_{{{{{{\rm{f}}}}}}}$). HAVDI binding (N-cadherin based adherens junctions) disturbed integrin clustering and decreased ${k}_{{{{{{\rm{on}}}}}}}$ in integrin binding to RGD on ECMs. Decreased ${k}_{{{{{{\rm{on}}}}}}}$ reduced the ${F}_{{{{{{\rm{trac}}}}}}}$ in actin filaments, and hence deformation of the viscoelastic nucleus (spring stiffness ${E}_{{{{{{\rm{N}}}}}}}$ and viscosity ${\eta }_{{{{{{\rm{N}}}}}}}$). **b** The model for variation of ${k}_{{{{{{\rm{on}}}}}}}$ with ECM stiffness (${E}_{{{{{{\rm{sub}}}}}}}$) on HAVDI/RGD (H/R) and Scram/RGD (S/R) substrates. **c** Traction on H/R or S/R substrates with different stiffness ${E}_{{{{{{\rm{sub}}}}}}}$, showing that H/R increased the stiffness threshold for generating elevated traction. Symbols are measured from experiments (mean ± s.e.m. from Supplementary Fig. 13b), while lines are modeling results. **d** In the force-dependent YAP redistribution model, elevated traction resulted in increased nuclear deformation, enlarged nuclear pores, increased YAP import rate, and thus higher YAP n/c ratio (${R}_{{{{{{\rm{NC}}}}}}}$) on HAVDI/RGD substrates than on Scram/RGD substrates. **e** The relationship between nuclear flattening (${\lambda }_{{{{{{\rm{N}}}}}}}$) and actin contraction. Symbols are measured from experiments (mean ± s.e.m. from Supplementary Fig. 14b), while lines are modeling results. **f** ${R}_{{{{{{\rm{NC}}}}}}}$ increased linearly with nuclear flattening for all culture conditions. The line is prediction by our model. Hollow squares are data from reference^31^. The values of solid symbols on *x*-axis (${\lambda }_{{{{{{\rm{N}}}}}}}$) are from Supplementary Fig. 14b. The values of solid symbols on *y*-axis (${R}_{{{{{{\rm{NC}}}}}}}$) are from Fig. 2f and Fig. 3c–e. The lateral and vertical error bars indicate s.e.m. Results indicated that ${R}_{{{{{{\rm{NC}}}}}}}$ is an indicator of nuclear deformation. **g** Validation of the model for cells cultured on substrates of different stiffness. The model reproduced the di-sigmoidal curves in data from S/R and H/R groups (3 d for culture time). The values of dots on *x*-axis (stiffness) are from Fig. 1c, The values of dots on *y*-axis (${R}_{{{{{{\rm{NC}}}}}}}$) are from Fig. 2f. The lateral and vertical error bars indicate s.e.m. **h** An explanation of mechanical memory in cells transferred from TCP (dark purple) to S/R (orange) or to H/R (blue) substrates, denoted TCP-S/R or TCP-H/R, respectively. ${R}_{{{{{{\rm{NC}}}}}}}$ increased with culture time due to plastic deformation of the nucleus. Differences between TCP-S/R and TCP-H/R groups could be attributed to the differences in elastic recovery of the nucleus modulated by N-cadherins. Source data are provided as a Source Data file.

Briefly, in the motor-clutch model (Fig. 5a), actin filaments attached to ECM through dynamic bonds at integrin clutches with binding rate (${k}_{{{{{{\rm{on}}}}}}}$) and unbinding rate (${k}_{{{{{{\rm{off}}}}}}}$), while myosin motors contracted the actin filaments with traction force (${F}_{{{{{{\rm{trac}}}}}}}$) at velocity (${v}_{{{{{{\rm{f}}}}}}}$). The actin filament deformed the nucleus and substrate simultaneously. The nucleus was regarded as viscoelastic, with elastic deformation that recovered transiently with reduced traction force and inelastic deformation that accumulated loading (culture) time on TCP. As expected, cell traction forces increased with substrate stiffness (${E}_{{{{{{\rm{sub}}}}}}}$) due to the tension-mediated recruitment of integrin, which resulted in a higher ${k}_{{{{{{\rm{on}}}}}}}$ on stiffer substrates^57^. The range of ${k}_{{{{{{\rm{on}}}}}}}$ depended upon the integrin density on the membrane^60^. N-cadherin could downregulate traction force and focal adhesion length over an intermediate range of ${E}_{{{{{{\rm{sub}}}}}}}$ (5–20 kPa)^39^, due to lower integrin density and lower ${k}_{{{{{{\rm{on}}}}}}}$^60^. In our model, an Arrhenius relationship was used to relate ${k}_{{{{{{\rm{on}}}}}}}$ and ${E}_{{{{{{\rm{sub}}}}}}}$ to the presentation of HAVDI (details in Supplementary Methods) (Fig. 5b). The different ${k}_{{{{{{\rm{on}}}}}}}-{E}_{{{{{{\rm{sub}}}}}}}$ relationships on Scram/RGD and HAVDI/RGD hydrogels resulted in double sigmoidal relationships between ${F}_{{{{{{\rm{trac}}}}}}}$ and ${E}_{{{{{{\rm{sub}}}}}}}\,$ (Fig. 5c). The stiffness threshold for elevating traction force was thus higher on HAVDI/RGD substrates than on Scram/RGD substrates.

Stress fibers alignments are well known to respond dynamically to the mechanics of the cellular microenvironment^61–63^. To validate model predictions, we first measured the organization of F-actin (quantified by F-actin anisotropy). In accordance with prior observations^39^, F-actin anisotropy increased with substrate stiffness, and decreased significantly in presence of HAVDI on substrates of intermediate stiffness (20 kPa) (Supplementary Fig. 12). We then performed traction force microscopy for cells cultured on substrates of prescribed peptide mixtures and stiffness. For cells seeded on Scram/RGD hydrogels, the traction increased with increasing hydrogel stiffness (Supplementary Fig. 13). For cells seeded on HAVDI/RGD hydrogels, consistent with observations of Cosgrove et al.^39^ and our models, tractions were reduced by HAVDI presentation on substrates with an intermediate range of substrate moduli (10–20 kPa), but unaltered for compliant (1 kPa) or stiff (41 kPa) substrates (Fig. 5c). These results verified the prediction that MSC mechanosensing, and the peptide dependence thereof, required actomyosin contractility.

The model predicted that ${R}_{{{{{{\rm{NC}}}}}}}$ increased with increasing nuclear deformation, due to previously reported increases in the rate of translocation of cytoplasmic YAP to the nucleus^31^. In our model, HAVDI/RGD and Scram/RGD substrates yielded different tractions, nuclear deformation, pore sizes and hence ${R}_{{{{{{\rm{NC}}}}}}}$ (Fig. 5d). We hypothesized that actomyosin contractility affects nuclear deformation, which in turn affects the YAP n/c ratio. We thus characterized the nuclear flattening (${\lambda }_{{{{{{\rm{N}}}}}}}$, nuclear length/height), a measure of nuclear deformation evidenced from our confocal images, and confirmed that it increased monotonically with increasing ${F}_{{{{{{\rm{trac}}}}}}}$ (Fig. 5e, Supplementary Fig. 14). As predicted, nuclear flattening was proportional to ${R}_{{{{{{\rm{NC}}}}}}}$ (Fig. 5f), an effect attributed to the enlargement of nuclear pores in deformed nuclei that promoted YAP import rate without changing the export rate^31^. Thus, ${R}_{{{{{{\rm{NC}}}}}}}$ served as a proxy for nuclear deformation in our model.

Our predictions of ${R}_{{{{{{\rm{NC}}}}}}}$ matched trends observed in our experiments, and were consistent with the model that HAVDI and stiffness co-determined ${k}_{{{{{{\rm{on}}}}}}}$, and that this effect gave rise to the observed double sigmoidal relationships between ${R}_{{{{{{\rm{NC}}}}}}}$ and ${E}_{{{{{{\rm{sub}}}}}}}$ on substrates with and without HAVDI, showing attenuated YAP nuclear localization (Fig. 5g). After this verification of the reasonability of our model for ${k}_{{{{{{\rm{on}}}}}}}$, the model was used to reproduce the phenomena observed in Fig. 3. Consistent with other observations of nuclear deformation, the nucleus was treated as viscoelastic, with deformation having both elastic (reversible) and plastic (irreversible) components^64–66^. Our model predicted the reductions in ${R}_{{{{{{\rm{NC}}}}}}}$ over time following transferal of MSCs from TCP to soft hydrogels, and attributed these trends to reductions in tractions associated with release of elastic deformation of the nucleus. The plastic deformation of the nucleus captured the observed increase in ${R}_{{{{{{\rm{NC}}}}}}}$ with increasing culture time on TCP (Fig. 5h). In cells transferred from TCP to soft gels, the elastic deformation recovered while the plastic deformation remained, reducing ${R}_{{{{{{\rm{NC}}}}}}}$ over time. ${R}_{{{{{{\rm{NC}}}}}}}$ in cells transferred to HAVDI/RGD substrates was lower than in those transferred to Scram/RGD substrates because HAVDI reduced traction and thus facilitated elastic recovery. Results suggested that effects of HAVDI on mechanical sensation and nuclear translocation of YAP observed in our culture system could be explained through motor-clutch dynamics.

MSCs in vivo receive mechanical cues from both cell-ECM and cell-cell adhesions, with the former promoting mechanosensing and the latter inhibiting it. We showed that these two antagonistic factors also affect nuclear translocation of YAP, and found that N-cadherin signaling can reverse these effects arising from cell-ECM adhesions. This is important for regenerative therapies^10^ involving in vitro expansion of MSCs on TCP and subsequent in vivo transplantation, which must overcome the effects of TCP exposure that bias MSCs toward osteogenic differentiation. Using PEG hydrogels with co-presentation of RGD and HAVDI peptides that we showed to mimic cell-ECM and cell-cell adhesions, we demonstrated that HAVDI signaling can be used to reverse a nuclear translocation of YAP, as well as sustained mechanosensing effects in the form of RUNX2, ALP and OCN activity by altering the motor-clutch dynamics in MSCs. Taken together, results show that N-cadherin signaling arising from cell-cell adhesion reduces long-term retention of the effects of mechanotransduction in MSCs and can be used to engineer cell culture platforms to modulate “mechanical memory” in hMSCs.

## Methods

### Preparation of PEG hydrogels and peptide conjugation

PEG hydrogels were prepared from 8-arm PEG maleimide (hexaglycerol) (PEG-MAL, 10 kDa, JenKem Technology) backbone and 8-arm PEG thiol (hexaglycerol) (PEG-SH, 10 kDa, JenKem Technology) crosslinker. RGD peptides (GCGYGRGDSSPG) for fibronectin, and HAVDI (HAVDIGGGC) or scrambled HAVDI control (AGVGDHIGC) peptides for N-cadherin (Sangon Biotech, Shanghai) were covalently conjugated to the PEG-MAL backbone via Michael addition reactions between the cysteine residues on these peptides and the maleimide on the PEG-MAL backbone. PEG-MAL (5 mM) and peptides were dissolved in phosphate-buffered saline (PBS) for 1 h at 37 °C for peptide conjugation. For these studies, all peptides were used at a final concentration of 1 mM in hydrogels (except for hydrogel control without any peptide conjugation).

### Hydrogel fabrication and characterization

Hydrogels with a final thickness of ~200 μm were formed after 30 min Michael addition reaction between dual peptide modified PEG-MAL and PEG-SH crosslinker on acrylated cover glasses (diameter ~14 mm). After multiple PBS washes (>4 times for 5 min each to remove the unreacted groups), the as-prepared hydrogels were used to characterize mechanical properties or for cell experiments. Rho-RGD (rhodamine-labeled RGD peptide) and FITC-HAVDI (FITC-labeled HAVDI peptide) were used to characterize the distributions and concentrations of RGD or HAVDI peptides in hydrogels and to determinate their coupling efficiency by measuring the fluorescence spectroscopy at 588 nm and 520 nm, respectively. Briefly, the prepared PEG hydrogels incorporated with 1 mM peptides (Rho-RGD or FITC-HAVDI) were rinsed with PBS ( > 4 times for 5 min each) to collect unbound peptides on a supernatant solution. The supernatant and the control solution (containing the initial peptide concentration of 1 mM) were both diluted with PBS to obtain the same volume. Their fluorescence emission intensity was measured to calculate the conjugation efficiency.

### Mechanical characterization of hydrogels

Indentation experiments were performed to estimate the Young’s moduli of hydrogels. Following standard procedures^67–69^, Young’s modulus was assessed from load-displacement curves obtained using a commercial nanoindenter (Piuma Nanoindenter, Optics11, Amsterdam, N.L.) with a microsphere indentation tip (diameter: 2.5 µm, stiffness: 0.28 N/m). Load-displacement curves (representative data are shown in Supplementary Fig. 3) were converted to load-indentation curves and fitted to the Hertz model, assuming a linear elastic and isotropic material response^70^:

1 $$P=\frac{4}{3}\frac{E}{1-{\upsilon }^{2}}{R}^{1/2}{h}^{3/2}$$

where $P$ is the load on the hydrogel, $R$ is the radius of the spherical tip, $h$ is the indentation depth, and $E$ and $\upsilon$ are the Young’s modulus and Poisson’s ratio of the hydrogel, respectively. For isotropic materials, including PEG hydrogels, the compressive and tensile moduli often have the same amplitude, and vary together with changes in material parameters^71^. Thus, the Young’s modulus obtained by localized indentation could reflect the substrate stiffness sensed by cells. For each condition, the mean and standard error of mean were obtained from dozens of random points on 3 hydrogel replicates. We note that, unlike most ECM materials, which are hyperelastic and/or fibrous in character^72–74^, the hydrogel used in this study was well fit by the Hertz model. Although this simplifies analysis, it is a limitation of the study and future work should address the role of ECM nonlinearity on binding energetics and kinetics.

### hMSC isolation

Human mesenchymal stem cells (hMSCs) were isolated from human bone marrow provided by commercial sources (Cyagen Biosciences). Briefly, cells were obtained from donors by bone marrow aspiration, then monocyte density centrifugation was performed and selected for adherent culture. Standard analytical methods were used to screen cell growth and differentiation into fat and bone.

### Cell culture

Isolated hMSCs (labeled as P1) from human bone marrow were frozen down in protein-free cryopreservation medium (Cyagen, HUXMX-07021) and then used for all experiments in this manuscript. Growth medium (Cyagen, HUXMA-90011) was changed every 2-3 days. Hydrogels for cell culture were sterilized in 75% (v/v) aqueous ethanol for 3–4 h followed by five rinses with sterilized PBS as described^75^. Prior to cell seeding, hydrogels were prewetted with growth medium for 30 min. hMSCs were seeded at low density (1000 cells per cm^2^) to avoid cell-cell interactions, except as noted. Sparse or confluent cells were seeded at 1000 or 20,000 cells per cm^2^. For studies without mechanical dosing on TCP, hMSCs were directly seeded on soft hydrogels after cell thawing. For studies with mechanical dosing on TCP, hMSCs were seeded on TCP at cell density from 1000 to 20,000 cells per cm^2^, depending on the culture time on TCP. For myosin inactivation studies, inhibition of myosin was achieved using 50 µM blebbistatin (Abcam, ab120425) for 30 min. For osteogenic differentiation studies, cells were cultured in an osteogenic medium (Cyagen, HUXMA-90021) to remove any confounding effect of chemical dosing and accelerate the process of osteogenic differentiation. For N-cadherin-blocking studies, 1 mM soluble HAVDI(-C) peptides (HAVDIGGG) were added to the growth media directly following hMSCs seeding, according to methods previously described^39,76^. The soluble peptides designed to bind with the N-cadherin receptor on the cell membrane can avoid conjugating to the PEG-MAL backbone by removing cysteine in the sequence of original HAVDI peptides.

### Immunostaining and quantification

hMSCs were fixed in 4% paraformaldehyde (formaldehyde solution: PBS = 1:9) for 20 min, followed by 10 min permeabilization with 0.5% Triton X-100 in PBS, and 30 min blocking non-specific binding sites with 5% bovine serum albumin (BSA) in PBS. Primary antibodies were diluted in 1% BSA in PBS and added overnight at 4 °C. Antibodies and dilutions used in this study contained anti-YAP (1:100, rabbit, Cell Signaling no. 14074), anti-β-catenin (1:200, mouse, Cell Signaling no. 2677), anti-N-cadherin (1:200, rabbit, Cell Signaling no. 13116), anti-RUNX2 (1:1600, rabbit, Cell Signaling no. 12556), anti-OCN (1:200, rabbit, Thermo Fisher no. PA5-96529). After three PBS rinses, AlexaFluor-488[H + L] secondary antibodies (1:500, goat anti-rabbit, Cell Signaling no. 4412) and AlexaFluor-647[H + L] secondary antibodies (1:500, goat anti-mouse, Cell Signaling no. 4410) were added for 2 h at room temperature, followed by F-actin staining using Rhodamine Phalloidin (1:1,000; Invitrogen no. R415) incubated for 30 min. All immunostained samples were embedded in ProLong® Gold Antifade Reagent with DAPI (Cell Signaling no. 8961) and visualized with Olympus FV3000 confocal microscope at 10 × 0.4NA (1.243 μm/pixel) and 60 × 1.42NA (0.207 μm/pixel) oil immersion objective lenses. The amount of the N-cadherin and β-catenin in a region of mimetic or real cell-cell adhesion was quantified utilizing RGB Profiler plugin in Image J. Percentages of OCN positive cells were calculated by counting the number of OCN positive cells and then dividing by the total number of cells based on DAPI staining. To exclude the data of adjacent cells with cell-cell interactions, only the immunostaining results of single cells having no contact with neighbors were counted for quantification in our study, except as noted the case of the confluent cells.

### ALP staining and quantification

hMSCs were fixed with 4% paraformaldehyde for 20 min, followed by washing with PBS, and stained with BCIP/NBT Alkaline Phosphatase Color Development Kit (Beyotime, C3206) following the manufacturer’s instructions. Cell nuclei were then stained with DAPI to count the total cell numbers. Percentages of ALP positive cells were calculated by counting the number of blue-violet stained cells and then dividing by the total cell number.

### YAP and RUNX2 n/c ratio quantification

For sparse cells, the nucleus and cytoplasm were identified by F-actin and DAPI staining, and the n/c ratio ${R}_{{NC}}^{{sparse}}$ for YAP or RUNX2 was calculated following techniques used by others^21,77–79^ as the ratio of the total fluorescence intensity in the nucleus, ${I}_{{nucleus}}$, to the total fluorescence in the remainder of the cell, weighted by the areas of the nucleus and the remainder of the cell:

2 $${R}_{{NC}}^{{sparse}}=\frac{({I}_{{nucleus}}/{A}_{{nucleus}})}{({I}_{{cell}}-{I}_{{nucleus}})/({A}_{{cell}}-{A}_{{nucleus}})}\,$$

where ${A}_{{nucleus}}$ is the area of the nucleus as measured by DAPI staining, ${A}_{{cell}}$ is the overall area of the cell as delineated by F-actin staining, and ${I}_{{cell}}$ is the total fluorescence intensity in the overall cell. Intensities and areas were measured using Image J (1.52p). Images of cells with saturated fluorescence were excluded.

For confluent cells, a multicellular zone of cells contacting with neighbors was delineated, and the above procedures were repeated to obtain the average YAP n/c ratio, ${R}_{{NC}}^{{confluent}}$, over the region of confluent cells:

3 $${R}_{{NC}}^{{confluent}}=\frac{\left(\mathop{\sum }\limits_{i=1}^{N}{I}_{{nucleus}}^{i}\right)\bigg/\left(\mathop{\sum }\limits_{i=1}^{N}{A}_{{nucleus}}^{i}\right)\,}{\left({I}_{{zone}}-\mathop{\sum }\limits_{i=1}^{N}{I}_{{nucle}{us}}^{i}\right)\bigg/\left({A}_{{zone}}-\mathop{\sum }\limits_{i=1}^{N}{A}_{{nucleus}}^{i}\right)\,}$$

where ${A}_{{nucleus}}^{i}$ is the area of the nucleus *i* in the zone containing *N* nuclei, ${I}_{{nucleus}}^{i}$ is the total fluorescence of nucleus *i*, ${I}_{{zone}}$ is the total fluorescence intensity of the zone, and ${A}_{{zone}}$ is the area of the zone.

### Nuclear deformation analysis

z-stacks of 0.5 µm steps were captured, nuclear x-y projections (major and minor axis of nucleus) and x-z projections were reconstructed using Bitplane Imaris (7.2.3) software on the DAPI channel to measure the nuclear length (major axis) and nuclear height, respectively. Nuclear flattening was calculated as the ratio of nuclear length to height^31^.

### Implementation of motor-clutch model

The model was modified from a well-established stochastic motor-clutch model^56–58,60^. Briefly, the model considered the connection between actin filament and substrate was realized through ${n}_{{{{{{\rm{c}}}}}}}$ clutches (focal adhesion complex, including integrin and adapter proteins). ${n}_{{{{{{\rm{m}}}}}}}$ myosin molecules with stall force (${F}_{{{{{{\rm{m}}}}}}}$) pulled the actin filament at a retrograde velocity ${v}_{{{{{{\rm{u}}}}}}}$. The clutches broke and reconnected according to characteristic on and off rates (${k}_{{{{{{\rm{on}}}}}}}$ and ${k}_{{{{{{\rm{off}}}}}}}$). ${k}_{{{{{{\rm{on}}}}}}}$ was dependent upon the integrin density on the cell membrane, which changed according to the conformation (folding or unfolding) of the adapter protein talin. Previous studies have shown that ${k}_{{{{{{\rm{on}}}}}}}$ grew with increasing substrate stiffness (${E}_{{{{{{\rm{sub}}}}}}}$)^60^. Here, the relationship between ${k}_{{{{{{\rm{on}}}}}}}$ and HAVDI presentation was modeled using Arrhenius function. Simulation ran for 10^5^ time steps (>1000 s) to ensure the reaching of a steady state. Details of the model can be found in Supplementary Methods and Supplementary Table 1. The calculation of the model is conducted by a custom code^80^.

### Statistical analysis

All data for cell experiments were collected using a single cell line studied on at least three hydrogel replicates per condition, except where noted. The number of cells counted for each condition was indicated in each figure legend. Statistical comparisons were performed with Graphpad Prism (8.0.1) or Origin (2020) using one-way analysis of variance (ANOVA) with Tukey’s post hoc test for comparison of multiple groups. Differences were considered to be significant when *p* values were below 0.05. Details of sample sizes and significance levels were given in figure legends. In all figures, data were shown as mean ± standard error of the mean (s.e.m.) unless otherwise stated.

### Reporting summary

Further information on research design is available in the Nature Research Reporting Summary linked to this article.

## Supplementary information

Supplementary Information

Reporting Summary

## Data Availability

Source data of the Figs. 1c, 2f, 3c-e, 4c, 4e, 4g, 5b, 5c, 5e, 5g, 5h; Supplementary Fig. 4, Supplementary Fig. 5, Supplementary Fig. 6b, Supplementary Fig. 7b, Supplementary Fig. 8b, Supplementary Fig. 9c, Supplementary Fig. 10b, Supplementary Fig. 11, Supplementary Fig. 12b, Supplementary Fig. 13b, Supplementary Fig. 14b-e are provided with this paper. All other relevant data supporting the key findings of this study are available within the article and its Supplementary Information files or from the corresponding author upon reasonable request. Source data are provided with this paper.

## Source data

Source Data

## Code availability

Source code for motor-clutch model is available for download at https://github.com/zhuhy2021/Motor-clutch-model-including-HAVDI-effect (10.5281/zenodo.5537041)^80^.
