# Supplementary material for: Mechanics-driven nuclear localization of YAP can be reversed by N-cadherin ligation in mesenchymal stem cells
Source: Nat Commun. 2021 Oct 28;12:6229. doi: 10.1038/s41467-021-26454-x (PMC8553821; doi:10.1038/s41467-021-26454-x)
Supplement: Supplementary file 1 — Supplementary Information [file 41467_2021_26454_MOESM1_ESM.pdf]

Supplementary information for "Mechanics-driven nuclear localization of YAP can be reversed by N-cadherin ligation in mesenchymal stem cells"

Cheng Zhang[1,2*], Hongyuan Zhu[1,2*], Xinru Ren[1,2], Bin Gao[3], Bo Cheng[1,2], Shaobao Liu[4], Baoyong Sha[5], Zhaoqing Li[1,2], Zheng Zhang[1,2], Yi Lv[6], Haohua Wang[6], Hui Guo[7], Tianjian Lu[4,8], Feng Xu[1,2], Guy M. Genin[1,2,9,10], Min Lin[1,2#]

1 The Key Laboratory of Biomedical Information Engineering of Ministry of Education, School of Life Science and Technology, Xi'an Jiaotong University, Xi'an 710049, P.R. China
2 Bioinspired Engineering and Biomechanics Center (BEBC), Xi'an Jiaotong University, Xi'an 710049, P.R. China
3 Department of Endocrinology, Second Affiliated Hospital of Air Force Military Medical University, Xi'an 710038, China
4 State Key Laboratory of Mechanics and Control of Mechanical Structures, Nanjing University of Aeronautics and Astronautics, Nanjing 210016, P.R. China
5 Institute of Basic Medical Science, School of Basic Medical Science, Xi'an Medical University, Xi'an 710021, China
6 National Local Joint Engineering Research Center for Precision Surgery & Regenerative Medicine, Shaanxi Provincial Center for Regenerative Medicine and Surgical Engineering, The First Affiliated Hospital of Xi'an Jiaotong University, China
7 Department of Medical Oncology, The First Affiliated Hospital of Xi'an Jiaotong University, Xi'an 710061, Shaanxi, China
8 MOE Key Laboratory of Multifunctional Materials and Structures, Xi'an Jiaotong University, Xi'an 710049, P.R. China
9 Department of Mechanical Engineering & Materials Science, Washington University in St. Louis, St. Louis 63130, MO, USA
10 NSF Science and Technology Center for Engineering Mechanobiology, Washington University in St. Louis, St. Louis 63130, MO, USA

* These authors contributed equally to this work.
# Corresponding author: minlin@xjtu.edu.cn

This file includes

Supplementary Figures 1-14

Supplementary Text

Supplementary Methods

Supplementary Table 1

Supplementary References

Supplementary Figures

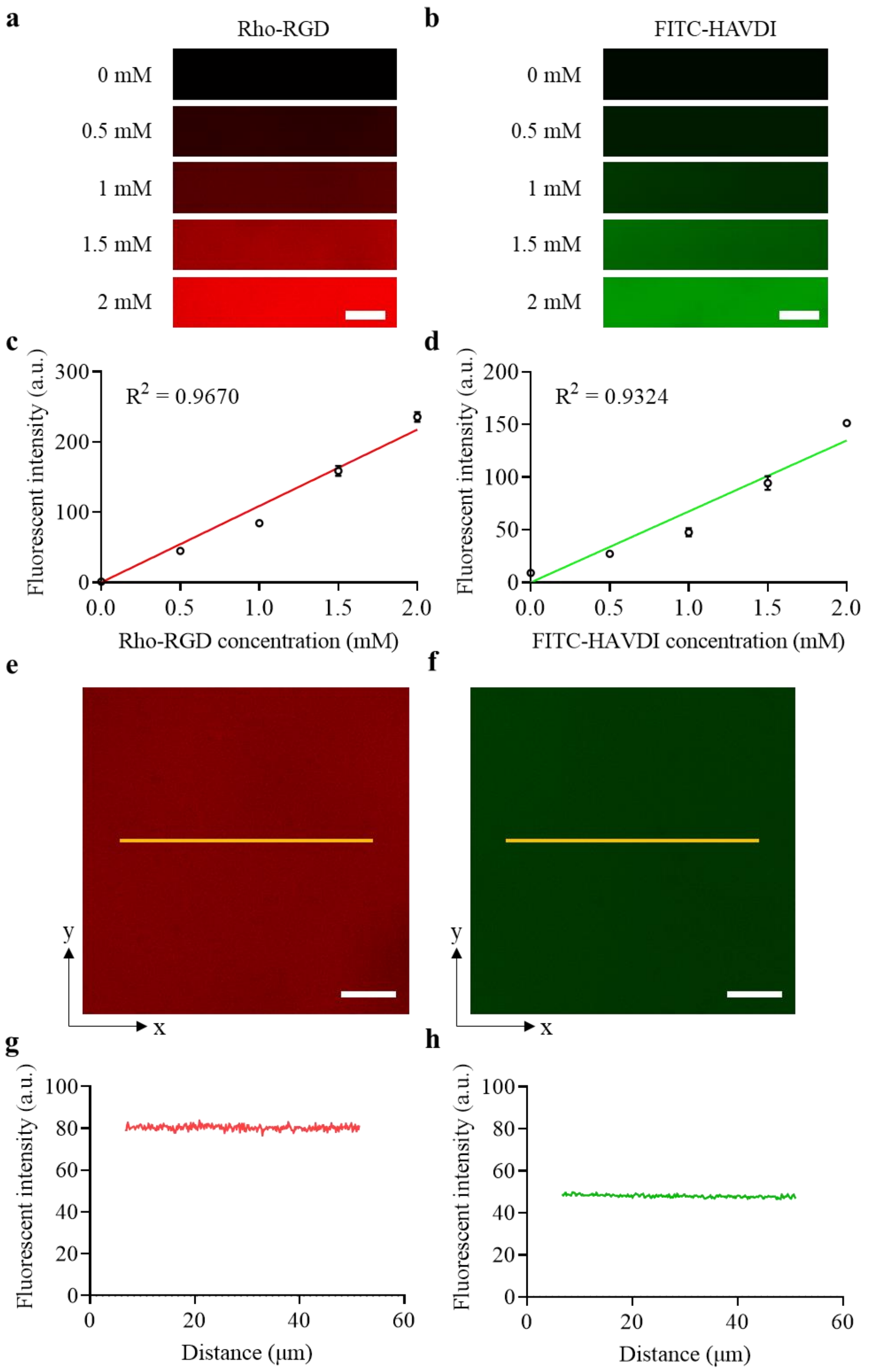

Supplementary Figure 1. Hydrogel fluorescence with different concentrations of fluorescent peptides. a-b, Representative fluorescence images for hydrogels with different concentrations of Rho-RGD (rhodamine-labeled RGD peptide, **a**), and

FITC-HAVDI (FITC-labeled HAVDI peptide, **b**). Scale bar: 100 μm. **c-d**, Quantification of fluorescence intensity of images in (**a**) and (**b**) respectively. (mean ± s.d., n = 111936 pixels adopted from 3 images for each group). The distance for one pixel is 1.243 μm. **e-f**, Representative images of fluorescence of hydrogels with 1 mM Rho-RGD (**e**) and FITC-HAVDI (**f**). Yellow lines indicate the pixel regions used to generate intensity profiles. **g-h**, Fluorescence intensity profiles on yellow lines shown in (**e**) and (**f**) respectively. The results showed that no peptide clustering in both groups. Scale bar: 100 μm.

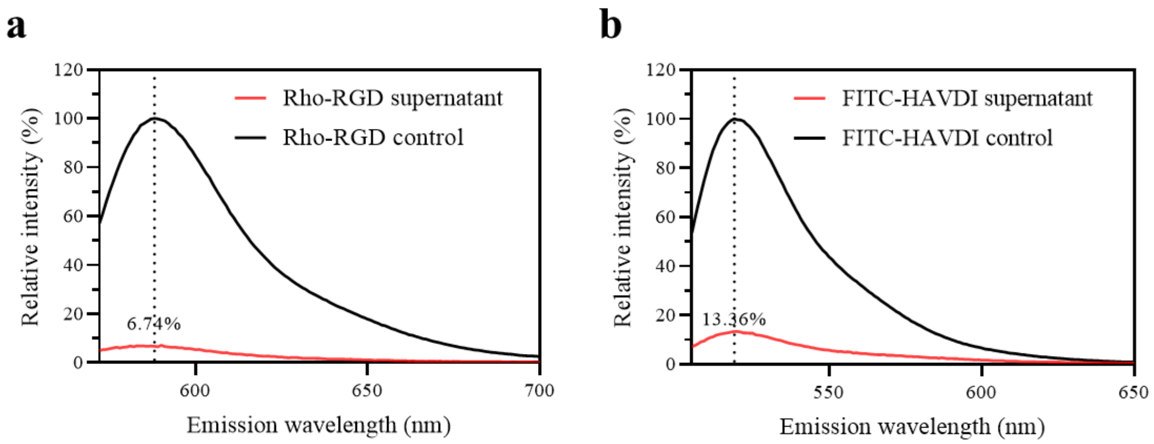

Supplementary Figure 2. Determination of the coupling efficiencies of (a) Rho-RGD (rhodamine-labeled RGD peptide) and (b) FITC-HAVDI (FITC-labeled HAVDI peptide) on a PEG-MAL backbone, as quantified by fluorescence spectroscopy. Fluorescence values of Rho-RGD and FITC-HAVDI were detected at 588 nm and 520 nm. Their coupling efficiencies were calculated through the difference of fluorescence intensities between the supernatant (6.74% in **a**, 13.36% in **b**) and control solution.

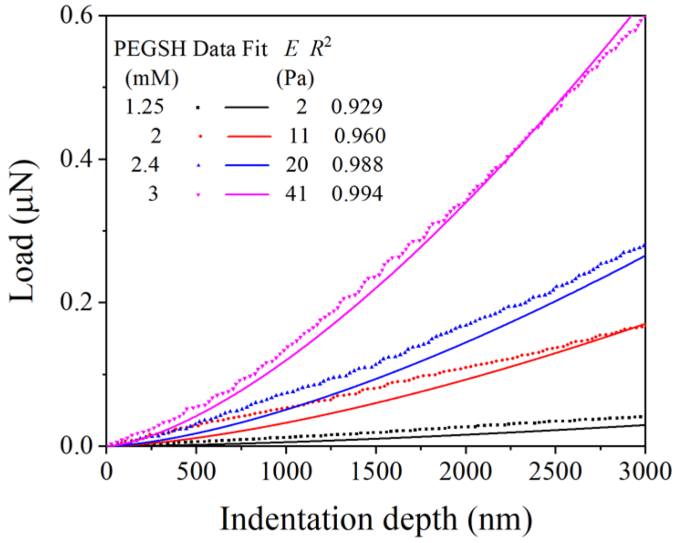

Supplementary Figure 3. **Representative load-indentation curves of PEG hydrogels synthesized with different initial concentrations of PEGSH.**

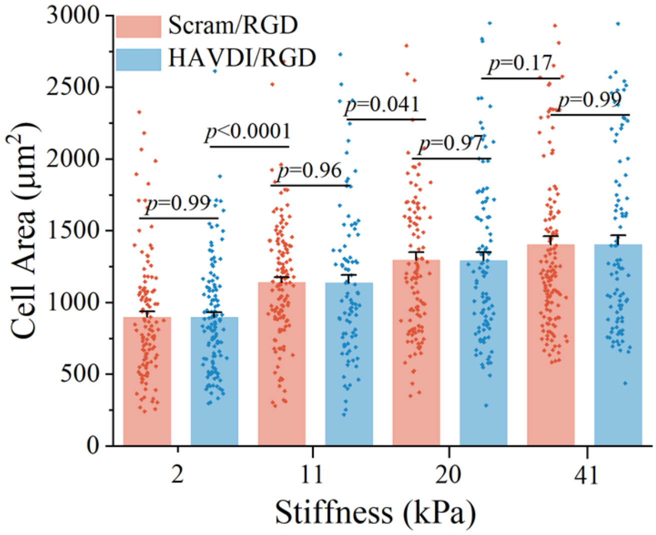

Supplementary Figure 4. hMSC spreading area on HAVDI/RGD or Scram/RGD hydrogels as a function of substrate Young's modulus. Cell spreading area increased with increasing substrate stiffness. But no significant difference in cell spreading area could be identified between the HAVDI/RGD and Scram/RGD groups. This indicated that the presentation of HAVDI did not alter cell spreading area in this system. Data were shown as mean ± s.e.m. (from left to right n = 138, 147, 129, 115, 121, 93, 88, 91 cells examined over 19, 20, 26, 17, 21, 18, 26, 21 images respectively, p values were obtained using two-way ANOVA followed by Tukey's post hoc test). Source data are provided as a Source Data file.

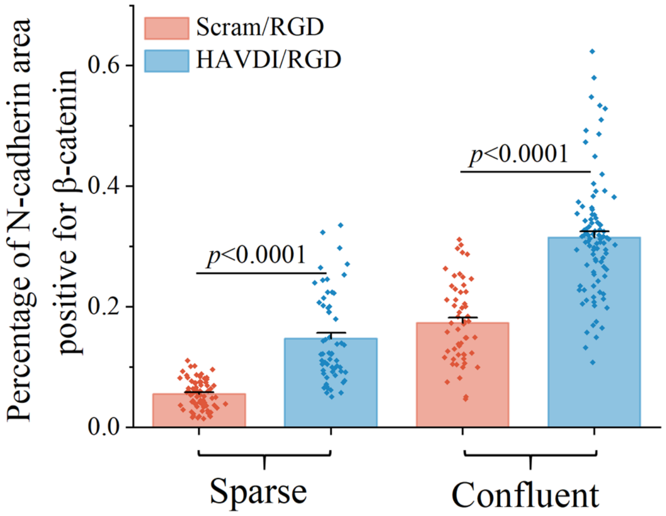

Supplementary Figure 5. Quantification of N-cadherin area positive for β-catenin shown in Figure 2a-d (from left to right n = 74, 58, 56, 92 cells examined over 21, 27, 21, 27 images respectively, p values were obtained using one-way ANOVA followed by Tukey's post hoc test, mean ± s.e.m.). Source data are provided as a Source Data file.

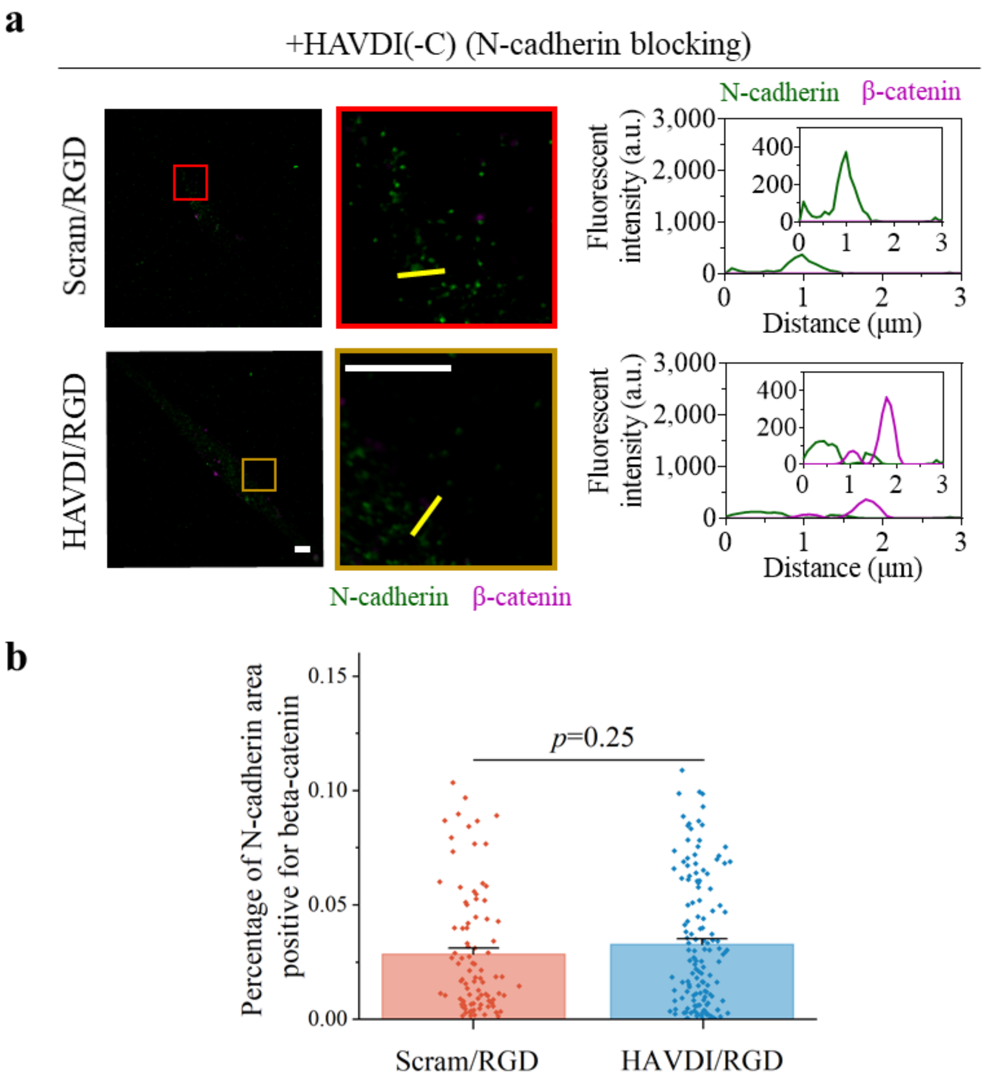

Supplementary Figure 6. Soluble HAVDI(-C) peptide inhibited N-cadherin clustering and β-catenin recruitment. a, Confocal images of N-cadherin and β-catenin subcellular distributions in hMSCs following the blocking of N-cadherin through addition of soluble HAVDI(-C) peptide. Yellow lines indicate the regions of interest (ROIs) used to generate intensity profiles of N-cadherin (green) and β-catenin (purple). hMSCs showed negligible N-cadherin clustering and β-catenin recruitment in both groups. Scale bars, 5 μm. **b,** Quantification of N-cadherin area positive for β-catenin (n = 92, 142 cells examined over 25, 36 images for Scram/RGD and HAVDI/RGD respectively, p values were obtained using one-way ANOVA followed by Tukey's post hoc test, mean ± s.e.m.). Source data are provided as a Source Data file.

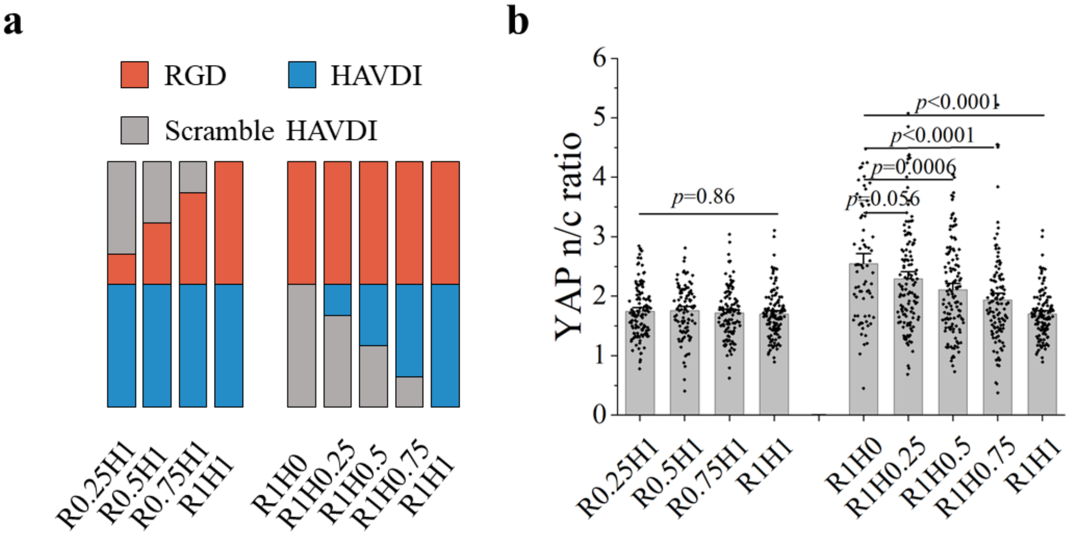

Supplementary Figure 7. The effect of RGD or HAVDI peptide concentrations on YAP nuclear localization. a, Schematic of RGD, HAVDI and Scramble HAVDI peptide concentrations on 20 kPa hydrogels while kept total effective ligand density as 2 mM. **b**, Quantification of YAP n/c ratios on 20 kPa hydrogels with increased RGD peptide and constant 1 mM HAVDI peptide (**left**) or with increased HAVDI peptide and constant 1 mM RGD peptide (**right**). (from left to right n = 122, 97, 119, 124, 71, 129, 126, 115, 124 cells examined over 13, 8, 6, 6, 15, 9, 9, 6, 6 images respectively, p values were obtained using one-way ANOVA followed by Tukey's post hoc test, mean ± s.e.m.). Source data are provided as a Source Data file.

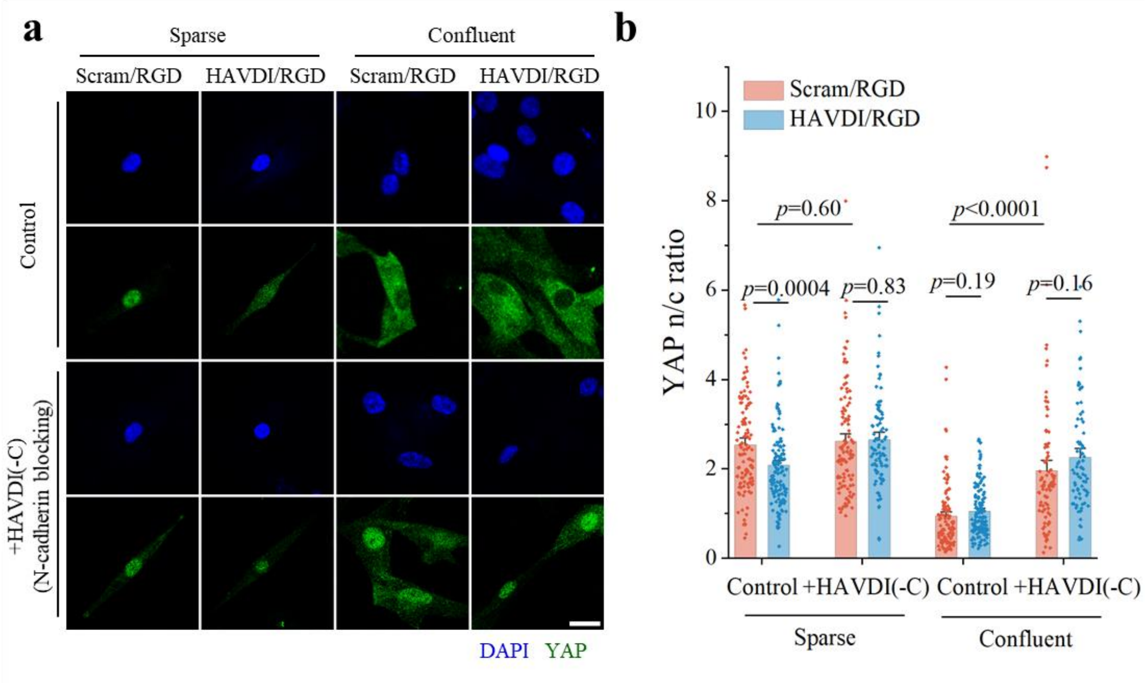

Supplementary Figure 8. YAP nuclear localization via immobilized HAVDI ligation induced N-cadherin signaling. **a**, Representative images of YAP immunostaining (green) in hMSCs, cultured as sparse or confluent cells on either Scram/RGD or HAVDI/RGD hydrogels, and followed by competition with either no peptide (Control) or 1 mM of soluble HAVDI(-C) peptide, which blocks N-cadherin binding, in growth media for 3 d. Scale bars, 20 µm. **b**, Quantification of YAP n/c ratios for conditions corresponding to panel (**a**). (from left to right n = 110, 135, 111, 91, 104, 138, 91, 77 cells examined over 4, 5, 5, 5 3, 4, 5, 5 images respectively, p values were obtained using one-way ANOVA followed by Tukey's post hoc test, mean ± s.e.m.). Sparse hMSCs on HAVDI/RGD hydrogels showed a significant increase in YAP n/c ratio when treated with N-cadherin blocking HAVDI(-C) peptide, as did confluent hMSCs on Scram/RGD. Source data are provided as a Source Data file.

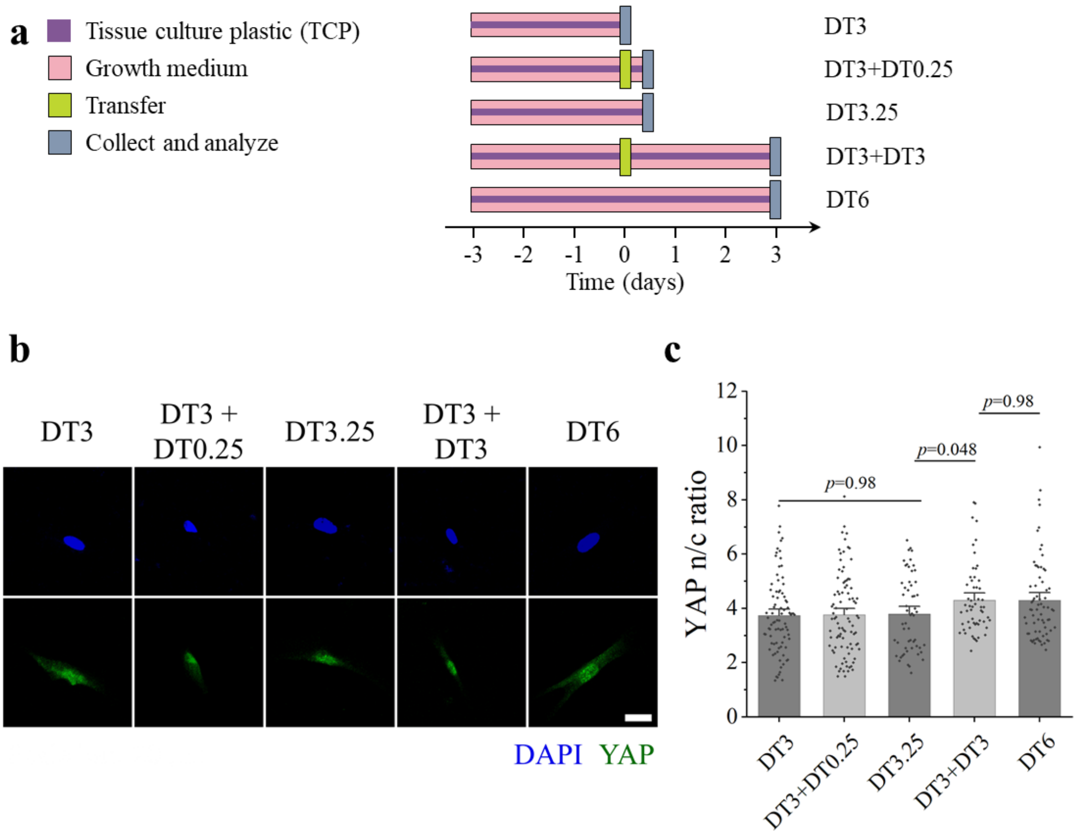

Supplementary Figure 9. YAP nuclear/cytoplasmic ratio in hMSCs cultured on TCP with or without trypsinization. a, Schematic of the experimental protocol for assessing the effect of trypsinization on YAP n/c ratio in hMSCs. With trypsinization, hMSCs were cultured on a TCP substrate (dark purple) in growth medium (pink) for 3 d following transferring (light yellow, day 0) to a different TCP substrate for an additional 0.25 d (6 h) or 3 d before collection and analysis (grey), denoted DT3+DT0.25 and DT3+DT3. Without trypsinization, hMSCs were cultured on TCP substrates (dark purple) in growth medium (pink) for 3, 3.25 or 6 d before collection and analysis (grey), denoted DT3, DT3.25 and DT6. **b-c**, Representative images (**b**) and quantification (**c**) of YAP staining in the indicated conditions shown in (**a**) (from left to right *n* = 84, 91, 56, 56, 69 cells examined over 8, 12, 10, 12, 15 images respectively, *p* values were obtained using one-way ANOVA followed by Tukey's post hoc test, mean ± s.e.m.). Scale bars, 20 μm. hMSCs showed no significant difference in YAP n/c ratios among DT3, DT3+DT0.25 and DT3.25 conditions, similar with the case between DT3+DT3 and DT6 conditions, indicating that trypsinization and transfer did not affect the YAP n/c ratio in a measurable way. Source data are provided as a Source Data file.

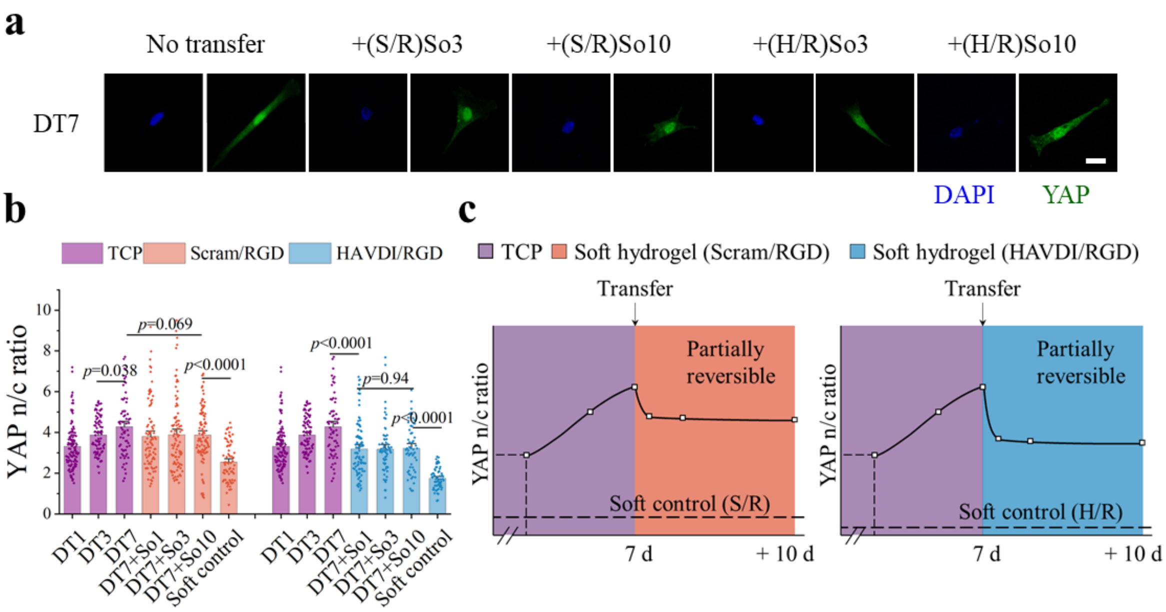

Supplementary Figure 10. YAP immunostaining of MSCs on Scram/RGD and HAVDI/RGD hydrogels after culturing on TCP for 7 d. a, Immunostaining of hMSCs cultured on TCP substrates for 7 d followed by transferring and 3 d culture on soft Scram/RGD or HAVDI/RGD hydrogels. Nucleus (blue) and YAP (green). Scale bars, 20 µm. S/R is Scram/RGD, H/R is HAVDI/RGD. **b**, Quantification of YAP n/c ratios in hMSCs cultured in conditions indicated in (**a**). (from left to right n = 108, 85, 66, 85, 93, 97, 71, 108, 85, 66, 93, 63, 57, 68 cells examined over 5, 6, 9, 37, 10, 29, 15, 5, 6, 9, 21, 23, 11, 13 images respectively, p values were obtained using one-way ANOVA followed by Tukey's post hoc test, mean ± s.e.m.). **c**, After 7 d of culture in TCP, YAP n/c ratios in both S/R and H/R groups decreased and remained above the baseline of soft control (S/R or H/R) (DT7+So1, 3, 10), while maintaining the significant difference in YAP n/c ratios between S/R and H/R groups. Source data are provided as a Source Data file.

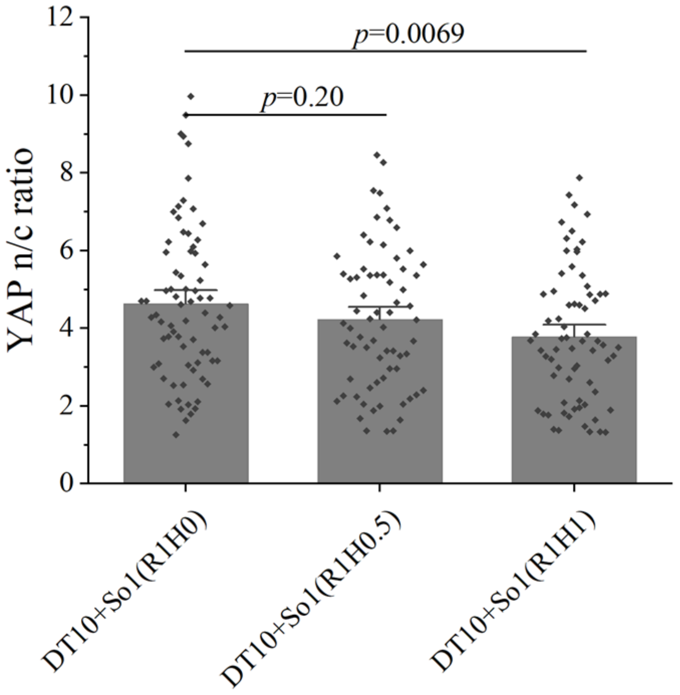

Supplementary Figure 11. Quantification of YAP nuclear/cytoplasmic (n/c) ratios in hMSCs on TCP substrates for 10 d followed by transferring and 1 d culture on 20 kPa hydrogels with increased HAVDI peptide (0, 0.5, 1 mM) and constant 1 mM RGD peptide. (from left to right n = 74, 69, 70 cells examined over 15, 13, 13 images respectively, p values were obtained using one-way ANOVA followed by Tukey's post hoc test, mean ± s.e.m.). Source data are provided as a Source Data file.

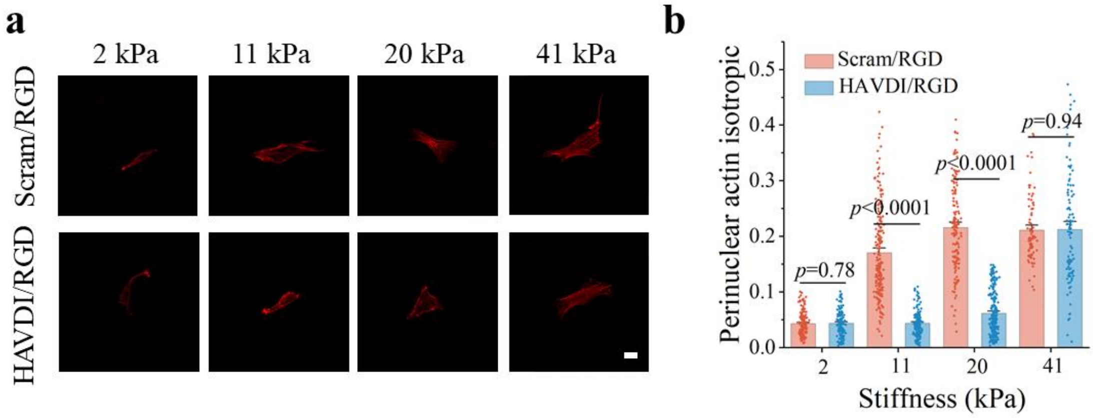

Supplementary Figure 12. HAVDI/N-cadherin interaction diminished actin organization at the medium stiffness. a, Representative images of F-actin immunostaining in hMSCs, cultured on 2, 11, 20 and 41 kPa Scram/RGD or HAVDI/RGD hydrogels. Scale bars, 20 µm. **b**, Quantification of actin organization and polarization via anisotropy ratios for conditions corresponding to panel (**a**). (from left to right n = 148, 141, 195, 144, 139, 231, 86, 100 cells examined over 16, 14, 17, 13, 12, 12, 8, 14 images respectively, p values were obtained using one-way ANOVA followed by Tukey's post hoc test, mean ± s.e.m.). Source data are provided as a Source Data file.

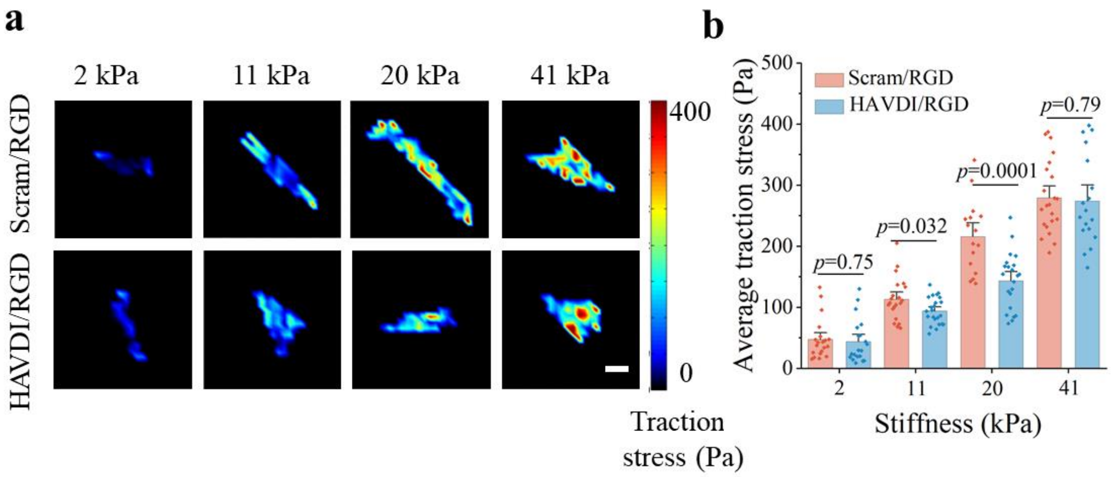

Supplementary Figure 13. HAVDI/N-cadherin interactions reduce the traction stress at the medium stiffness. a, Representative heat maps of traction stress in hMSCs, cultured on 2, 11, 20 and 41 kPa Scram/RGD or HAVDI/RGD hydrogels. Scale bars, 20 μm. **b**, Quantification of average traction stress per cell for conditions corresponding to panel (**a**). (from left to right n = 20, 20, 21, 23, 16, 22, 22, 18 cells examined over 17, 16, 16, 17, 10, 17, 19, 8 sets of images respectively, p values were obtained using one-way ANOVA followed by Tukey's post hoc test, mean ± s.e.m.). Source data are provided as a Source Data file.

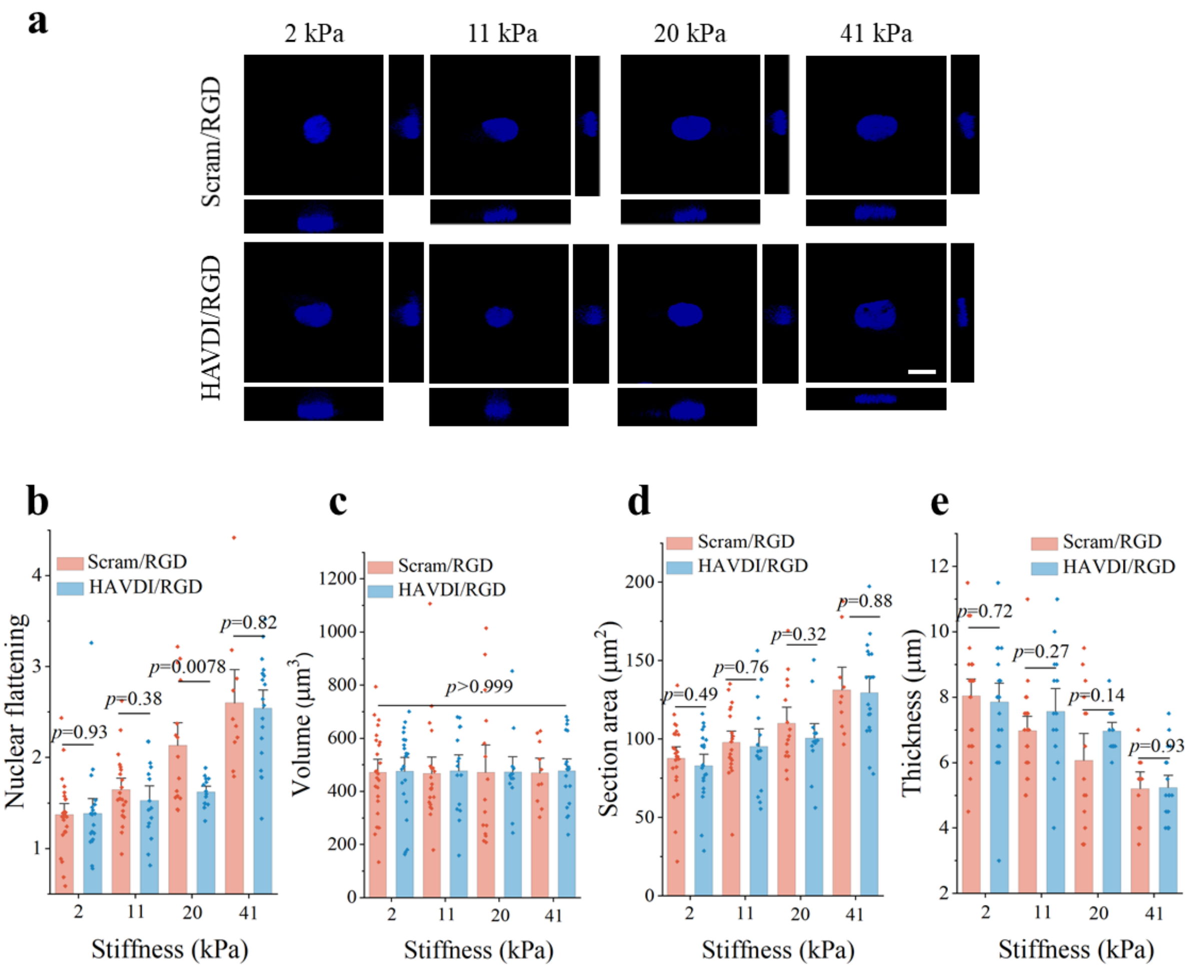

Supplementary Figure 14. HAVDI/N-cadherin interaction inhibited nuclear deformation. a, Representative images of DAPI immunostaining in hMSCs, cultured on 2, 11, 20 and 41 kPa Scram/RGD or HAVDI/RGD hydrogels. Scale bars, 10 μm. **b-e**, Quantification of nucleus via nuclear flattening (**b**), nuclear volume (**c**), section area (**d**) and nuclear thickness (**e**), and for conditions corresponding to panel (**a**). (from left to right n = 24, 21, 21, 15, 15, 14, 10, 19 cells examined over 18, 14, 14, 12, 9, 7, 10, 13 images respectively, p values were obtained using one-way ANOVA followed by Tukey's post hoc test, mean ± s.e.m.). Source data are provided as a Source Data file.

Supplementary Text

To quantify the distributions and surface concentrations of peptides grafted onto PEG hydrogels, fluorescence labels were grafted onto peptides (red rhodamine for RGD, and green FITC for HAVDI) to visualize peptides (**Supplementary Fig. 1**). For both RGD and HAVDI, fluorescence intensity increased linearly with the concentration of incubated peptide concentration, indicating that the surface peptide concentration was proportional to the incubated peptide concentration (**Supplementary Fig. 1c-d**). The relatively low standard deviation of pixel fluorescence intensity for each group suggesting that peptide clustering on the surfaces of the PEG hydrogels was minimal (**Supplementary Fig. 1e-h**).

Assuming a uniform distribution of peptides, a coupling efficiency (β) and an incubated concentration (C_{i}), we could evaluate the surface concentration (C_{s}) of the peptides on the hydrogels. The derivation follows: (i) the peptide concentration remaining in the hydrogels is $C_{\mathrm{i}}\beta$ (mol·L^{-1}); (ii) assuming that the peptides are arranged in a cubic lattice, the distance between the two neighboring peptide molecules is $d = 0.1/\sqrt[3]{C_{\mathrm{i}}\beta N_{\mathrm{A}}}$ (m), where N_{A} is Avogadro's constant; (iii) assuming the surface peptide to be a single molecular layer, the surface concentration (C_{s}) could be calculated by:

$$C_{\mathrm{s}} = C_{\mathrm{i}}\beta d = 10^2 N_{\mathrm{A}}{}^{-1/3}(C_{\mathrm{i}}\beta)^{2/3} \ \ (\mathrm{mol \cdot m^{-2}}) \tag{1}$$

Since the coupling efficiencies of 1 mM RGD and HAVDI were measured as 93.3% and 86.4% respectively (**Supplementary Fig. 2**), the corresponding distances between the neighbor peptide molecules are 12.1 and 12.4 nm, and the corresponding surface concentrations are 6830 and 6504 molecules·μm^{-2}. Fibronectin polymers in a natural matrix contribute one epitope per protein copy that measures several nanometers [1]. A critical RGD nanospacing of 70 nm has been proposed, beyond which a sharp decrease of cell adhesion was observed [2, 3]. This suggested that the peptide density we used fell within a suitable regime.

Supplementary Methods

F-actin analysis. The degree of F-actin organization (quantified by F-actin anisotropy) based on rhodamin-phalloidin staining was calculated by a FibrilTool plugin in Image J [4].

Traction Force Microscopy (TFM). Traction force microscopy was performed according to previous descriptions [5]. Fluorescent microspheres (Invitrogen, F8810) with a diameter of 0.2 μm were incorporated into the PEG hydrogel precursor (dual peptide modified PEG-MAL) at a final concentration of 1% v/v, prior to hydrogel formation. PEG hydrogels for TFM experiments were formed after the Michael addition reaction between the fluorescent microspheres incorporated hydrogel precursor and PEG-SH cross-linker. After multiple PBS washes (> 4 times for 5 min each to remove the unreacted groups), hydrogels were sterilized in 75% (v/v) aqueous ethanol for 3-4 h followed by five rinses with sterilized PBS as described [6]. hMSCs were seeded at 1,000 cells per cm^2 on PEG hydrogels and allowed to culture for 1 d before performing TFM analysis. Fluorescent images of multiple cells and embedded microspheres were captured at 40× magnification on an Olympus FV3000 confocal microscope. Image sequences of each cell, taken before and after lysis of the cells with RIPA lysate, were analyzed by a previously published MATLAB script [7] to obtain a traction force vector map and the average traction stress exerted per cell on the underlying substrate.

Model assumptions and implementation

Our model is modified from the stochastic motor-clutch model developed by David Odde et al. [8]. In the model, n_{m} myosin motors pull an actin filament with a force (F_{m}) at a velocity (v_{f}). The molecular clutch (a focal adhesion complex including integrin and adaptor proteins) reversibly engages to the actin filament with binding (k_{on}) and unbinding (k_{off}) rates and exerts traction force (F_{trac}) on the substrate. During contraction, the ith clutch is stretched to a strain $x_{\mathrm{clu}}(i)$, and local substrate is stretched to a strain, x_{sub}. The mechanical resistance of the clutch and substrate are determined by their spring constants (κ_{clu} and κ_{sub}) respectively.

1. Conversion relationship between spring constant and stiffness of substrate

Analog to previous work [9], the spring constant (κ_{sub}) of substrate is proportional to the substrate stiffness (E_{sub}) as:

$$E_{\mathrm{sub}} = \frac{9\kappa_{\mathrm{sub}}}{4\pi a} \tag{2}$$

where a is radius of focal adhesion.

2. Integrin-RGD binding and unbinding rate

Cellular force transmission and transduction can arise from a dynamic actin-talin-integrin-substrate clutch mechanism [10]. We modeled three key features of such interactions: (i) the integrin-ECM binding rate (k_{on}) is increases with integrin density and substrate stiffness (E_{sub}) [9, 10], and (ii) integrin density is related to focal adhesion length and to binding rates [11]; and (iii) the mechanics of cell-ECM interactions is governed by the enthalpy of attachment, as in the McEvoy-Desphande-McGarry model of the thermodynamics of cell spreading [12, 13]. Because a reduced focal adhesion length arises from HAVDI ligation [14], we hypothesized that the presentation of immobilized HAVDI reduced integrin density by reducing k_{on} due to the introduction of an energy barrier to binding, δU, over the natural energy barrier, so that:

$$\begin{aligned} k_{\mathrm{on}} &= A_0 \exp\left(-\frac{U_0(E_{\mathrm{sub}}) - U_{\mathrm{S/R}} + \delta U}{k_{\mathrm{B}}T}\right) \\ &= k_{\mathrm{on}}^{\mathrm{max}} \exp\left(-\frac{U_0(E_{\mathrm{sub}}) + \delta U}{k_{\mathrm{B}}T}\right) \end{aligned} \tag{3}$$

where A_0 is a constant, k_{B} is Boltzmann's constant, T is absolute temperature, $U_0 = b/(E_{\mathrm{sub}} + c)$ is the elastic contribution to the energy barrier (in reverse proportion to the substrate modulus E_{sub} and independent of surface peptides, b and c are fitting parameters), $U_{\mathrm{S/R}}$ is the energy of binding to an S/R hydrogel, and δU increases with the concentration of HAVDI, $k_{\mathrm{on}}^{\mathrm{max}}$ is the maximum value of k_{on}.

Similar to previous work [8, 15], the integrin-RGD unbinding rate was treated as a slip bond in response to the force (F_{clu}) on each integrin-RGD clutches as:

$$k_{\mathrm{off}} = k_{\mathrm{off0}} \exp(F_{\mathrm{clu}}/F_{\mathrm{b}}) \tag{4}$$

where F_b is characteristic rupture force of integrin-RGD bond. Although the bond between integrin and its most ligands show the catch-slip bond behavior [16], the simplified slip bond model could produce quantitatively similar fitting results [15].

3. Determination of traction force on a single actin filament

At each simulation time step, unbound molecular clutches were linked to the actin filaments according to the binding rate (k_{on}), while the bound molecular clutches are tested for breaking according to unbinding rate (k_{off}). Then value of force applied to the substrate (F_{sub}) generated by a single actin filament was calculated according to two relationships. The first one is the force balance between the substrate and integrin-RGD clutches:

$$F_{\mathrm{sub}} = \frac{\kappa_{\mathrm{sub}}\kappa_{\mathrm{clu}}\sum_{i=1}^{n_{\mathrm{bound}}} x_i}{\kappa_{\mathrm{sub}} + n_{\mathrm{bound}}\kappa_{\mathrm{clu}}} \tag{5}$$

where n_{bound} is the total number of bound integrin-RGD, x_i is the position of ith integrin-RGD clutch. The second one is the force-velocity relationship of actin filament:

$$v_{\mathrm{f}} = v_{\mathrm{u}}\left(1 - \frac{F_{\mathrm{sub}}}{n_{\mathrm{m}}F_{\mathrm{m}}}\right) \tag{6}$$

where v_{f} and v_{u} are actual and unloaded actin speed respectively. The simulation is allowed to run for 100,000 time steps (>1000 s) to ensure a steady-state before statistics. The traction force (F_{trac}) provided by actin filament is calculated as the mean value of F_{sub} over these time steps.

4. Nuclear deformation

The nuclear was considered as a viscoelastic material with elasticity (E'_{N}) and viscosity (η'_{N}), then the nuclear strain (ε_{N}) could be calculated by:

$$\varepsilon_{\mathrm{N}} = \left(\frac{1}{E'_{\mathrm{N}}} + \frac{t}{\eta'_{\mathrm{N}}}\right)\frac{F_{\mathrm{trac}}}{S_{\mathrm{N}}} = \left(\frac{1}{E_{\mathrm{N}}} + \frac{t}{\eta_{\mathrm{N}}}\right)F_{\mathrm{trac}} \tag{7}$$

where S_{N} is the binding area between a actin filament and the nuclear, E_{N} and η_{N} are effective elasticity and viscosity, respectively, regardless of the binding area.

When stretched by F_{trac}, the nuclear will be flattened (cross section is enlarged, while the thickness is compressed). The relationship between nuclear flattening (λ_{N}) and ε_{N}

is:

$$\lambda_{\mathrm{N}} = \sqrt{\frac{1+\varepsilon_{\mathrm{N}}}{1-\upsilon_{\mathrm{N}}\varepsilon_{\mathrm{N}}}} \tag{8}$$

where υ_{N} is the Poisson's ratio of nuclear.

5. The relationship between YAP nuclear/cytoplasm ratio with nuclear flattening

Similar to work by Pere Roca-Cusachs et al. [17], we found that there existed a linear relationship between YAP nuclear/cytoplasm ratio (R_{NC}) and nuclear flattening (λ_{N}) in all our experimental groups. Thus,

$$R_{\mathrm{NC}} = p\lambda_{\mathrm{N}} + q \tag{9}$$

Supplementary Table 1. Model parameters

Notation	Variable	Value	Unit	Ref
n_{c}	Number of integrin-RGD clutches	75	-	Adjusted
n_{m}	Number of myosin motors	75	-	Adjusted
F_{m}	Single myosin motor stall force	2	pN	[8]
v_{u}	Unloaded myosin motor velocity	110	nm/s	[11]
δU	Additional energy barrier to bind rate k_{on} introduced by 1mM immobilized HAVDI peptide	$1.31 \cdot 10^{-21}$	J	Adjusted
$k_{\mathrm{on}}^{\max}$	Maximum value of k_{on}	0.57	s^{-1}	Adjusted
k_{B}	Boltzmann's constant	$1.38 \cdot 10^{-23}$	J/K	-
T	Absolute temperature	310	K	-
k_{off0}	Unloaded integrin-RGD bond off-rate	0.1	s^{-1}	[8]
F_{b}	characteristic rupture force of integrin-RGD bond	6	pN	Adjusted
κ_{c}	Integrin-RGD clutch spring constant	0.8	pN/nm	[15]
a	Radius of adhesion	50	nm	[11]
ν_{N}	Poisson's ratio of nuclear	0.5	-	Adjusted
E_{N}	The effective elasticity of nucleus regardless of the actin-nuclear binding area respectively	101	pN	Adjusted
η_{N}	The effective viscosity of nucleus regardless of the actin-nuclear binding area respectively	$3.89 \cdot 10^{8}$	pN·s	Adjusted
p	The fitting parameter for the linear relationship between YAP nuclear/cytoplasm ratio and nuclear flattening	1.15	-	Adjusted
q	The fitting parameter for the linear relationship between YAP nuclear/cytoplasm ratio and nuclear flattening	-0.38	-	Adjusted

Supplementary References

1. Freeman R*, et al.* Instructing cells with programmable peptide DNA hybrids. *Nat. Commun.* **8**, 15982 (2017).
2. Arnold M*, et al.* Activation of integrin function by nanopatterned adhesive interfaces. *ChemPhysChem* **5**, 383-388 (2004).
3. Huang J*, et al.* Impact of Order and Disorder in RGD Nanopatterns on Cell Adhesion. *Nano Lett.* **9**, 1111-1116 (2009).
4. Boudaoud A*, et al.* FibrilTool, an ImageJ plug-in to quantify fibrillar structures in raw microscopy images. *Nat. Protoc.* **9**, 457-463 (2014).
5. Driscoll Tristan P, Cosgrove Brian D, Heo S-J, Shurden Zach E, Mauck Robert L. Cytoskeletal to Nuclear Strain Transfer Regulates YAP Signaling in Mesenchymal Stem Cells. *Biophys. J.* **108**, 2783-2793 (2015).
6. Ding B*, et al.* Tough and Cell-Compatible Chitosan Physical Hydrogels for Mouse Bone Mesenchymal Stem Cells in Vitro. *ACS Appl. Mater. Inter.* **8**, 19739-19746 (2016).
7. Butler JP, Tolić-Nørrelykke IM, Fabry B, Fredberg JJ. Traction fields, moments, and strain energy that cells exert on their surroundings. *Am. J. Physiol. Cell Ph.* **282**, C595-C605 (2002).
8. Chan CE, Odde DJ. Traction Dynamics of Filopodia on Compliant Substrates. *Science* **322**, 1687-1691 (2008).
9. Elosegui-Artola A*, et al.* Rigidity sensing and adaptation through regulation of integrin types. *Nat. Mater.* **13**, 631-637 (2014).
10. Elosegui-Artola A*, et al.* Mechanical regulation of a molecular clutch defines force transmission and transduction in response to matrix rigidity. *Nat. Cell Biol.* **18**, 540 (2016).
11. Oria R*, et al.* Force loading explains spatial sensing of ligands by cells. *Nature* **552**, 219-224 (2017).
12. McEvoy E, Deshpande VS, McGarry P. Free energy analysis of cell spreading. *J. Mech. Behav. Biomed.* **74**, 283-295 (2017).
13. McEvoy E, Shishvan SS, Deshpande VS, McGarry JP. Thermodynamic Modeling of the Statistics of Cell Spreading on Ligand-Coated Elastic Substrates. *Biophys. J.* **115**, 2451-2460 (2018).
14. Cosgrove BD*, et al.* N-Cadherin Adhesive Interactions Modulate Matrix Mechanosensing And Fate Commitment of Mesenchymal Stem Cells. *Nat. Mater.* **15**, 1297 (2016).
15. Bangasser Benjamin L, Rosenfeld Steven S, Odde David J. Determinants of Maximal Force Transmission in a Motor-Clutch Model of Cell Traction in a Compliant Microenvironment. *Biophys. J.* **105**, 581-592 (2013).
16. Kong F, García AJ, Mould AP, Humphries MJ, Zhu C. Demonstration of catch bonds between an integrin and its ligand. *J. Cell Biol.* **185**, 1275-1284 (2009).
17. Elosegui-Artola A*, et al.* Force Triggers YAP Nuclear Entry by Regulating Transport across Nuclear Pores. *Cell* **171**, 1397-1410 (2017).
